# An electroactive platform enabled by near-field communication for accelerating infected diabetic wound healing via directional electric field reshaping and immunomodulation

**DOI:** 10.1093/rb/rbag115

**Published:** 2026-06-09

**Authors:** Yuange Zong, Ying Chen, Kexin Deng, Peng Zheng, Hongling Zhou, Ze Zhang, Wanqi Huang, Danyang Huang, Yuan Peng, Jiaping Zhang

**Affiliations:** Department of Plastic Surgery, State Key Laboratory of Trauma and Chemical Poisoning, Southwest Hospital, Army Medical University (Third Military Medical University), Chongqing 400038, China; Department of Plastic Surgery, State Key Laboratory of Trauma and Chemical Poisoning, Southwest Hospital, Army Medical University (Third Military Medical University), Chongqing 400038, China; Department of Plastic Surgery, State Key Laboratory of Trauma and Chemical Poisoning, Southwest Hospital, Army Medical University (Third Military Medical University), Chongqing 400038, China; Department of Plastic Surgery, State Key Laboratory of Trauma and Chemical Poisoning, Southwest Hospital, Army Medical University (Third Military Medical University), Chongqing 400038, China; Department of Plastic Surgery, State Key Laboratory of Trauma and Chemical Poisoning, Southwest Hospital, Army Medical University (Third Military Medical University), Chongqing 400038, China; Department of Plastic Surgery, State Key Laboratory of Trauma and Chemical Poisoning, Southwest Hospital, Army Medical University (Third Military Medical University), Chongqing 400038, China; Department of Plastic Surgery, State Key Laboratory of Trauma and Chemical Poisoning, Southwest Hospital, Army Medical University (Third Military Medical University), Chongqing 400038, China; Department of Plastic Surgery, State Key Laboratory of Trauma and Chemical Poisoning, Southwest Hospital, Army Medical University (Third Military Medical University), Chongqing 400038, China; Department of Plastic Surgery, State Key Laboratory of Trauma and Chemical Poisoning, Southwest Hospital, Army Medical University (Third Military Medical University), Chongqing 400038, China; Department of Plastic Surgery, State Key Laboratory of Trauma and Chemical Poisoning, Southwest Hospital, Army Medical University (Third Military Medical University), Chongqing 400038, China

**Keywords:** near-field communication, bioelectric fields, infected diabetic wounds, electroactive hydrogels, intrinsic self-antibacterial

## Abstract

Electrical stimulation for diabetic wound healing predominantly relies on wired power supplies, which not only increase the risk of secondary infection and complicate clinical operation but may also misalign with the endogenous bioelectric field (EF), collectively limiting effective tissue repair. To address the above limitations, we developed a portable hydrogel dressing wirelessly powered by near-field communication (NFC) through mobile phones, which produces electrical stimulation in precise alignment with the EF and thus effectively facilitates diabetic wound repair. Mechanistic studies reveal that the reconstructed directional biomimetic electric field significantly activates the Phosphatidylinositol 3-kinase/AKT (PI3K/AKT) signaling pathway within the wound tissue microenvironment, thereby reshaping the immune response pattern and restoring immune homeostasis. Simultaneously, the hydrogel dressing efficiently promotes the migration and proliferation of epidermal cells and induces neovascularization, providing essential structural support for wound repair. By integrating wireless NFC technology with directional bioelectric field therapy, this study provides a novel paradigm for the repair of clinically refractory infected diabetic wounds and demonstrates significant value for clinical translation and application.

## Graphical abstract

**Graphical Abstract:**
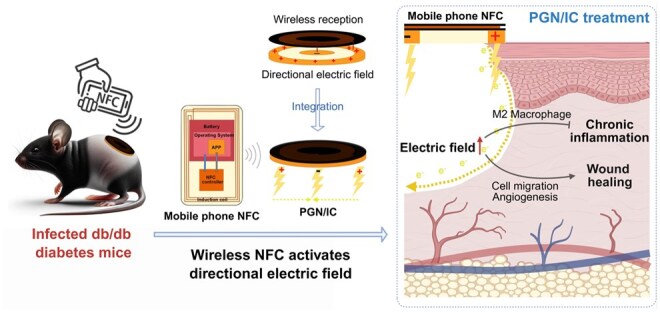

## Introduction

Following wound formation, a stable endogenous radial electric field is rapidly established at the wound margin via instantaneous alterations in epidermal transmembrane potential [1–3], which extends directionally from the peripheral wound edge toward the wound center (the peripheral region is positive, while the wound center is negative) [4–6]. As a core biological signal governing wound repair, endogenous electric fields directly mediate the directional proliferation and migration of key reparative cells such as fibroblasts and vascular endothelial cells, acting as a crucial driving force for physiological wound healing [7–9]. Furthermore, mounting recent studies have verified that EF can precisely sculpt the local wound inflammatory microenvironment by regulating pivotal processes including macrophage polarization and inflammatory cytokine secretion, thereby averting excessive or persistent inflammation and laying a stable immune microenvironment foundation for efficient wound repair [10–12].

In the diabetic pathological microenvironment, the intensity of the endogenous radial electric field at the wound site is significantly attenuated, accompanied by a drastically elevated risk of bacterial infection and chronic persistent inflammatory responses at the wound [13–15]. This ultimately impairs the directional proliferation and migration of wound repair-related cells, leading to wound ulceration [1, 16]. Although numerous studies have confirmed that exogenous electrical stimulation can effectively accelerate diabetic wound repair by compensating for the insufficient endogenous electric field [17, 18], current electrical stimulation devices employed in clinical practice and experimental research still present distinct limitations. Traditional devices are predominantly constructed with a wired power-supply framework [19, 20], which is cumbersome in operation [8, 21], elevates the risk of secondary infection owing to direct contact between leads and the wound surface [22, 23] and restricts patients’ limb mobility. By contrast, existing wireless batteryless devices, despite being liberated from physical cable restraints, necessitate frequent removal for recharging, which readily gives rise to intermittent disruptions in device function and data recording. Furthermore, their dependence on external wearable power modules tends to reduce patient compliance during long-term application. Collectively, these inherent drawbacks severely impede their clinical translation and widespread application [24].

In response to these challenges, our research team has achieved a series of important advances recently [25]. First, we developed an electrogenerative dressing (EGD) integrating a triboelectric nanogenerator with negative-pressure wound therapy, which enables the synergy of negative-pressure drainage and self-powered directional electric field stimulation [26]. This system can reshape the endogenous electric field weakened by negative pressure and regulate the local immune microenvironment of the wound [4, 27]. Second, we constructed a microneedle-based self-powered transcutaneous electrical stimulation system (mn-STESS) by combining a sliding free-standing triboelectric nanogenerator with a drug-loaded microneedle patch [28]. This system not only improves the transdermal permeability of drugs but also upregulates the expression of epidermal growth factor (EGF) receptors via electrical stimulation, thereby significantly enhancing the pharmaceutical efficacy of drugs and the wound repair effect [29]. These novel self-powered electrical stimulation intervention strategies have broken through the technical bottlenecks of traditional wired devices [30], providing a new direction for the treatment of diabetic wounds [31–33]. Yet, there remain several unsolved issues in the current research of such strategies [32, 34], such as the lack of precise directional control over electric fields [32, 35], and the urgent need to improve the antibacterial-electrical stimulation synergistic efficacy for infected diabetic wounds [36]. All the above-mentioned issues have become the key factors restricting the clinical translation of these novel intervention strategies [37].

**Figure 1:**
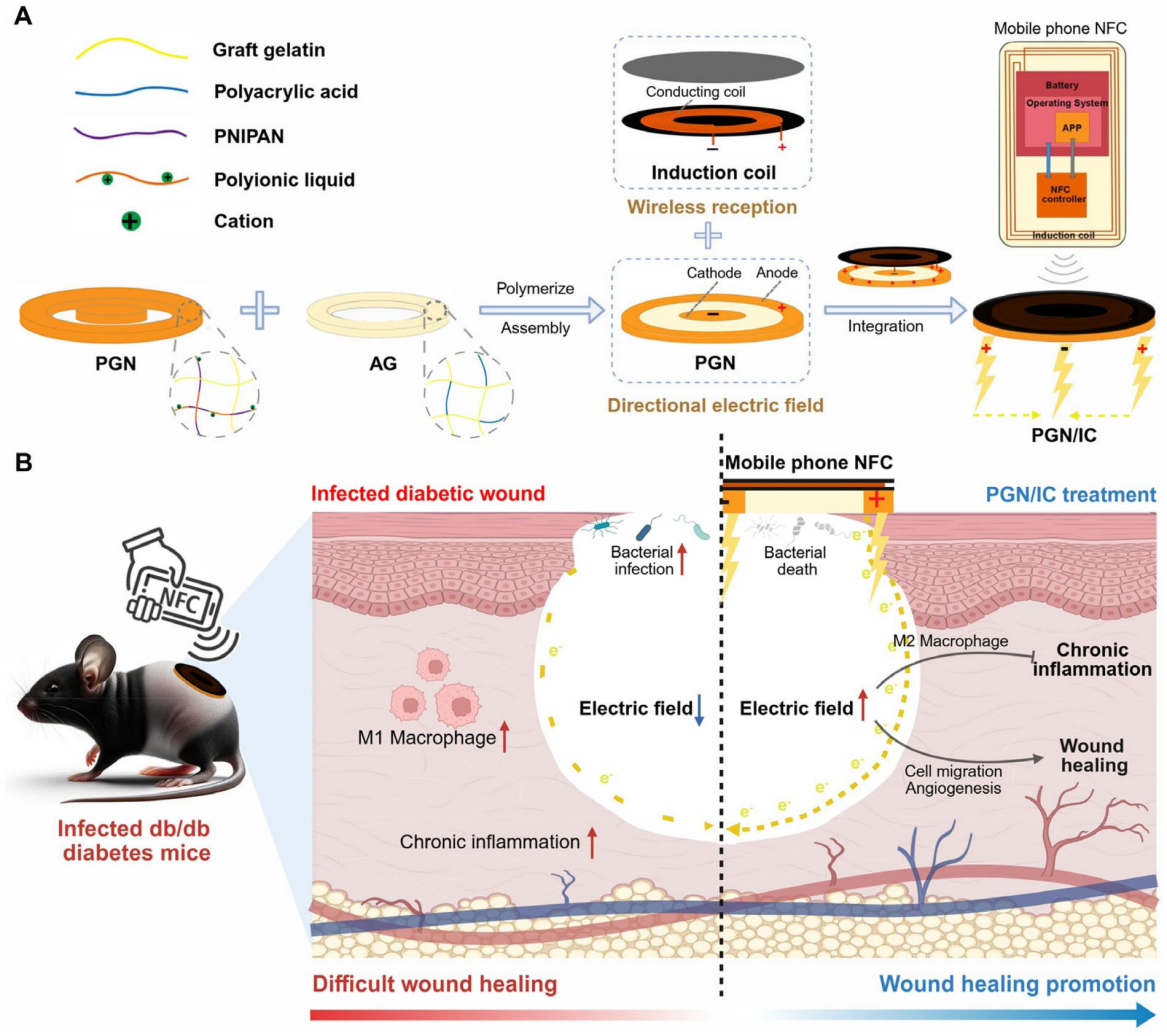
The schematic diagram shows: (**A**) schematic representation of the fabrication of PGN/IC electroactive hydrogels (EH) and the generation of stimulated electric field induced by NFC; (**B**) The mechanism of PGN/IC in regulating infected diabetic wound healing: remodeling the directional bioelectric field to accelerate angiogenesis, synergizing with broad-spectrum antibacterial efficacy to modulate the local inflammatory microenvironment, and ultimately promoting wound repair.

In this study, we integrated wireless near-field communication (NFC) power supply technology with electroactive hydrogels (EH) to construct PGN/IC, a novel wireless self-powered hydrogel platform employed as a functional dressing for infected diabetic wounds. Relying on its broad-spectrum antibacterial activity, this platform reconstructs a directional bioelectric field at the wound site and modulates the immune microenvironment, thereby accelerating wound healing. As illustrated in Figure 1A, PGN/IC possesses a sophisticated bilayer architecture: the upper layer serves as a wireless signal receiving module composed of an induction coil sandwiched between two polymer films, which captures NFC signals emitted by smartphones and converts them into direct current (DC) to power the entire system. The lower layer adopts a unique concentric-circle sandwich configuration, where the inner and outer rings are conductive PGN hydrogels (composited of polyionic liquid (PIL), gelatin and *N*-isopropylacrylamide (NIPAM)) connected to the negative and positive electrodes, respectively, and the interspace is filled with non-conductive AG hydrogels (prepared from polyacrylic acid (PAA) and modified gelatin) as an electrical insulation barrier. This elaborate design not only achieves effective spatial isolation between the anodic and cathodic rings but also generates a directional electric field in the wound area that precisely matches the human EF, mimicking the physiological repair microenvironment to promote tissue regeneration. When the PGN/IC patch was applied to infected wounds of db/db diabetic mice, it adhered tightly to the skin. DC was uniformly transmitted from the upper layer to the wound periphery, generating a centripetal outward current that effectively reconstructed a directional endogenous electric field at the wound site (Figure 1B). The reconstructed electric field acts synergistically with the broad-spectrum antibacterial properties of PGN/IC to jointly regulate the wound healing process, specifically manifested as accelerated M2 macrophage polarization, promoted epidermal cell proliferation and migration, and enhanced angiogenesis.

## Materials and methods

### Materials

Gelatin (∼250 bloom) was purchased from Shanghai Maokang Biotechnology Co., Ltd. 4-(4,6-Dimethoxytriazine-2-yl)-4-methylmorpholine hydrochloride, 4-bromomethylbenzaldehyde and NIPAM were provided by Shanghai Macklin Biochemical Technology Co., Ltd. Ethylenediamine, acetonitrile and acrylic acid were purchased from Sinopharm Chemical Reagent Co., Ltd. 1-Vinylimidazole was purchased from Shanghai Haohong Biomedical Technology Co., Ltd. Cell Counting Kit-8 and Calcein/PI Cell Activity and Cytotoxicity Test kits were purchased from Beyotime. E. coli (ATCC 25922), S. aureus (ATCC 25923) and MRSA (BNCC 337 371) were purchased from the China Microbiological Culture Preservation Center. Six-week-old male db/db mice were purchased from Chongqing Ensville Biotechnology Co., Ltd.

### Evaluation of wireless power transmission performance

The NFC induction coil module adopts a planar double-layer spiral coil structure, fabricated by laser etching of copper foil on a flexible polyimide (PI) substrate. The detailed parameters are as follows: total coil diameter: 20 mm; number of turns: 18 turns per layer, 36 turns in total; line width: 0.2 mm; line spacing: 0.2 mm; copper foil thickness: 35 μm; PI substrate thickness: 50 μm; inductance value: 4.7 μH; series resistance: 1.2 Ω; resonant frequency: 13.56 MHz, consistent with the standard NFC frequency of commercial smartphones. The coil is connected with a full-bridge rectifier circuit and a filter capacitor to convert the received alternating current (AC) into stable direct current (DC) output.

#### Working principle

NFC wireless power transmission is based on the principle of electromagnetic inductive coupling. When the NFC function of the smartphone is activated, the transmitting coil in the phone generates an alternating electromagnetic field at 13.56 MHz. The receiving induction coil in PGN/IC couples with the alternating magnetic field to generate induced electromotive force, which is converted into stable DC output through the rectifier and filter circuit and then applied to the concentric circular hydrogel electrodes to generate a directional electric field.

#### Operating conditions

The optimal operating distance between the smartphone transmitting coil and the PGN/IC receiving coil is 0–7 mm, with a maximum transmission distance of 10 mm. The input power of the smartphone NFC is 0.5 W, and the output voltage of the receiving coil can be stably adjusted in the range of 0.5–2.0 V.

To characterize the transmission performance of the wireless power module integrated in the PGN/IC hydrogel dressing, an NFC-based wireless power transfer system was employed, which comprised an induction transmitter and an induction receiver. The receiver was connected to a programmable DC power supply with adjustable output (range: 0.5–2 V, step: 0.1 V) to set and maintain its operating voltage, while the output voltage of the transmitter was simultaneously monitored using a digital multimeter to record power delivery characteristics under varying load conditions. For stability testing, the receiver voltage was first fixed at a target value (e.g. 1.0 V). The separation distance between the transmitter and receiver was then incrementally increased. At each distance, the system was allowed to stabilize for 30 s before recording the actual voltage received. These measurements enabled systematic analysis of NFC power transmission efficiency and stability across different separation distances and load settings, thereby providing critical parameters for subsequent *in vitro* and *in vivo* EF applications.

### Cell migration assay

The cell migration assay was performed on HaCaT cells. The extracted primary HaCaT cells were cultured in 1640 medium (Servicebio, G4538) containing 10% fetal bovine serum (Gibco) and 1% penicillin/streptomycin (Servicebio, G4003). After 24 h, the cells were transferred to a modified petri dish, and PGN/IC was used to power up the cells through mobile phone NFC technology. Cell migration was observed within 4 h using a Leica DMI6000 B living cell workstation, and the migration distance was measured using ImageJ software. To explore the mechanism through which the hydrogel promoted cell migration, Western blotting was used to detect the expression of proteins in the PI3K/AKT pathway.

### *In vivo* healing of infected wounds in db/db mice

All animal experiments were performed in accordance with the Guide for the Care and Use of Laboratory Animals of The First Affiliated Hospital of Army Military Medical University (Southwest Hospital) and were approved by the Institutional Ethics Committee of Experimental Animal Welfare Committee, PLA Army Medical University. The operator has passed the professional technical examination for laboratory animals (No. TY20201031). All animals in the experiments were in good condition according to the requirements of the National Laboratory Animal Law (China).

Eight-week-old male db/db mice with congenital diabetes mellitus were employed to establish the infected wound model. Prior to wound creation, the mice were anesthetized via intraperitoneal injection of pentobarbital sodium (40 mg/kg). After shaving and sterilizing the dorsal skin with 75% ethanol, a full-thickness excisional wound (diameter: 10 mm) was created using a sterile biopsy punch. Immediately thereafter, 200 μL of a standardized S. aureus suspension (approximately 1 × 10^8^ CFU/mL, prepared in sterile phosphate-buffered saline) was uniformly applied to the wound bed to establish a clinically relevant bacterial infection. The inoculated site was left uncovered for 30 min to allow bacterial adherence before the hydrogel dressing was applied. Wound healing progression was monitored through photographs captured on Days 0, 4, 8, 12 and 16 post-injury and infection. Wound areas were quantified using ImageJ software (NIH, USA). The wound healing rate was calculated according to the following formula: (A_0_ − A_t_)/A_0_ × 100%, where A_0_ represents initial wound area (Day 0) and A_t_ represents wound area at a specific time point (t).

On Day 4 post-injury, wound tissues were harvested, homogenized and subjected to quantitative bacterial culture. The bacterial load was determined through CFU counting. To explore the mechanism underlying the therapeutic effects of the hydrogel on the infected wounds, the expression levels of PI3K/AKT pathway–related proteins in the regenerating wound tissues were analyzed by Western blotting.

### Measurement of endogenous electric field at skin wounds in db/db mice

The distribution of endogenous EFs spontaneously generated at skin wounds in db/db diabetic mice was evaluated using a modified oscilloscope equipped with custom-designed metal probes, which enabled noninvasive measurement of potential differences. Prior to measurement, the metallic probe of the oscilloscope was partially insulated, leaving only the exposed front contact to minimize interference from ambient stray currents. During detection, the anode was positioned just outside the wound margin (approximately 1 mm from the edge), while the cathode was placed at the central region inside the wound, forming an EF loop across the lesion.

Mice were anesthetized, and the dorsal skin was prepared under sterile conditions before creating a full-thickness circular wound (diameter ≈ 6 mm) using a sterile biopsy punch. Immediately after wound formation (without application of any external dressing or Electrical stimulation), the modified probe was connected to the oscilloscope channel to monitor and record the potential difference waveform and amplitude across the wound in real time. The sampling rate was set to 1 kHz, and data were continuously acquired for 5 min to ensure stability. To reduce electromagnetic interference, all measurements were performed inside a shielded chamber, and baseline readings were obtained from healthy skin or unwounded regions under identical conditions for comparison.

### Statistical analysis

All experiments were repeated at least three times independently. All results are expressed as mean ± standard deviation (SD). The normality of the data was verified by the Shapiro-Wilk test, and the homogeneity of variance was verified by the Levene test. For comparisons among multiple groups, one-way analysis of variance (one-way ANOVA) was used to evaluate the statistical significance, followed by Tukey’s post-hoc multiple comparison test for pairwise comparisons between groups. For comparisons between two groups, the unpaired two-tailed Student’s t-test was used. The significance level was set as: *P < 0.05, **P < 0.01, ***P < 0.001, ****P < 0.0001; ns indicates no significant difference. All statistical analyses were performed using SPSS 22.0 software (IBM, USA), and graphs were plotted using GraphPad Prism 10.0 software.

## Results

### Preparation and characterization of PGN/IC

This study structurally optimizes previously reported EHs to construct a novel composite hydrogel system featuring gelatin as the main backbone [38, 39], supplemented with trace amounts of PAA, PIL and NIPAM (a thermosensitive monomer) [40–42]. As a natural, biodegradable biomaterial approved by the U.S. Food and Drug Administration [43–45], gelatin confers excellent biocompatibility and provides a robust structural backbone [46]. The introduction of trace amounts of PAA enhances the composite hydrogel’s mechanical stability and ion-crosslinking capacity [47]. The thermosensitive phase-change properties of NIPAM enable the considered dressing to actively contract at body temperature [48], thereby exerting traction on the wound bed margins to promote wound closure [49].

In PGN/IC, the upper layer is mainly responsible for receiving wireless transmission and providing power to the lower layer. The “concentric-circle sandwich” hydrogel in the lower layer is mainly responsible for uniformly applying a directional EF to the wound surface and providing continuous antibacterial activity. While preparing this hydrogel, we employed ethylenediamine-modified gelatin (Gela-NH_2_) as the main framework (Supplementary Figure S1). This framework exhibits excellent biocompatibility, laying the foundation for the safe application of this hydrogel to chronic wounds. Further, 4-(1 h-vinylimidazole)-1-methylene benzoic acid, an imidazole-based IL, was introduced into the system in a trace amount and subsequently copolymerized with NIPAM via Schiff-base and amidation reactions (Supplementary Figure S2). This approach preserves the inherent excellent biocompatibility of gelatin and markedly enhances the hydrogel’s ionic conductivity and thermosensitive shrinkage capacity [50].

Fourier transform infrared (FTIR) spectroscopy was employed to study the characteristic peaks of gelatin and Gela-NH_2_. The changes observed in the characteristic peaks at 1685 and 3464 cm^−1^ correspond to changes in the carboxyl (–COOH) and amino (–NH_2_) functional groups, respectively (Supplementary Figure S3). These changes are attributed to the grafting of ethylenediamine onto the main gelatin chain through amide bonds, increasing the proportion of the –NH_2_ groups, reducing the proportion of the –COOH groups and facilitating the chemical crosslinking of Gela-NH_2_. IL synthesis has been demonstrated in our previous research [51, 52]. PGN can function as a skin interface electrode owing to the skin-like conductivity of the IL within its structure (Supplementary Video S1).

**Figure 2:**
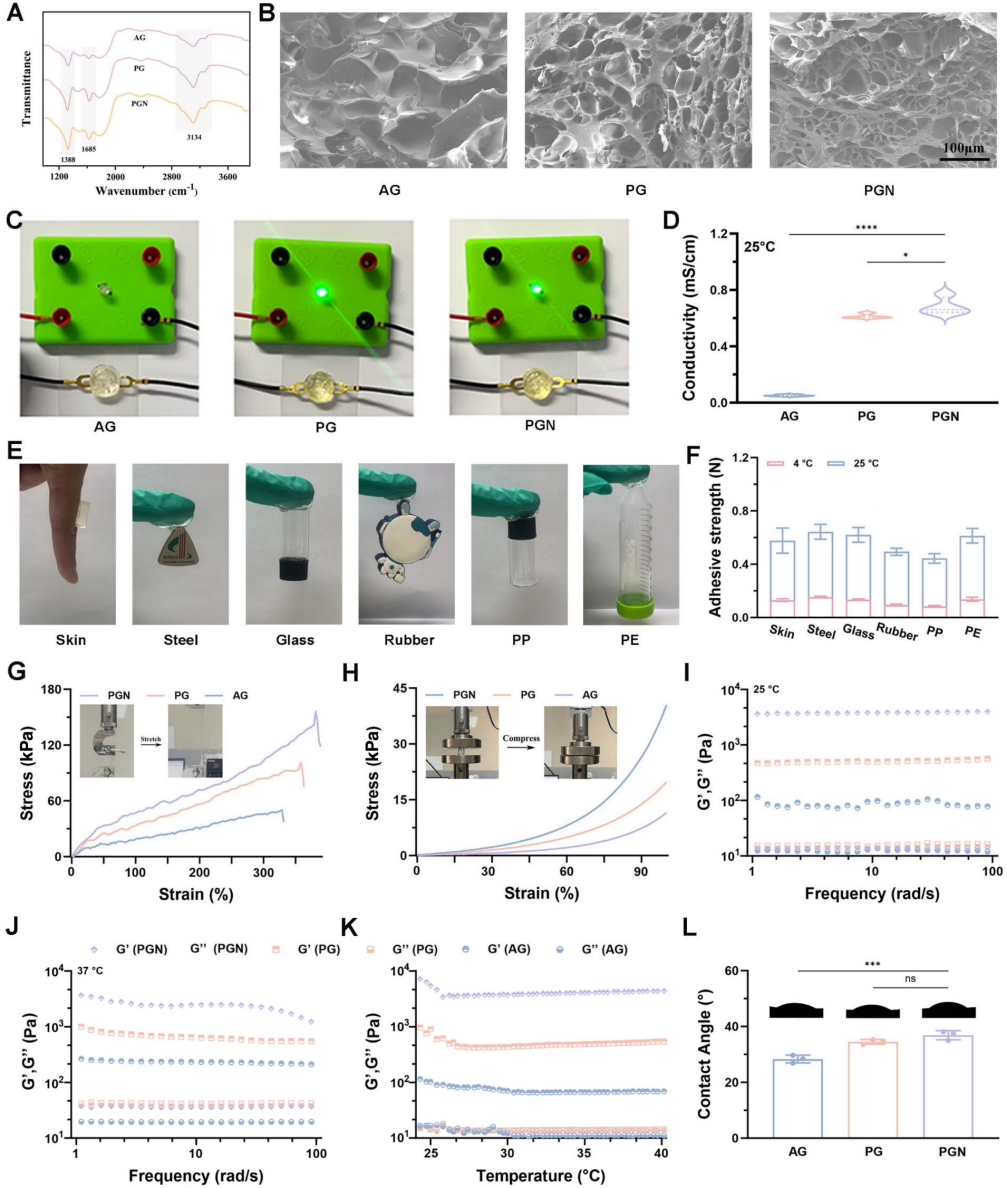
Preparation and physical characterization of hydrogels. (**A**) Fourier infrared spectra of a series of hydrogels. (**B**) SEM image of the internal structure of the hydrogel. Scale bar: 100 μm. (**C, D**) Optical images and conductivity statistics of hydrogels serving as conductors to light an LED bulb at 25°C. (**E, F**) Representative images and adhesion statistics of PGN hydrogel at room temperature (25°C) and low temperature (4°C). (**G**) Tensile stress of PGN, PG and AG hydrogels. (**H**) Compression stress of PGN, PG and AG hydrogels. (**I, J**) Frequency dependence of storage modulus (G′) and loss modulus (G″) for AG, PG and PGN hydrogels at 25 and 37°C. (**K**) Temperature sweep of G′ and G″ for PGN hydrogel (25–40°C). (**L**) Contact angle of hydrogels. *n* = 3 independent samples, data represent mean ± SD; *P < 0.05, **P < 0.01, ****P < 0.0001.

The characteristic peaks of a series of hydrogels were analyzed using FTIR spectroscopy (Figure 2A). The peaks at 1388, 1685 and 3134 cm^−1^ correspond to Schiff-base and amide bonds formed during gelation. Compared with AG, the addition of IL and NIPAM gradually resulted in additional peaks in the spectra of PG and PGN, indicating the formation of new chemical bonds. The introduction of PIL and NIPAM notably enhanced the crosslinking density of the hydrogel. This structural evolution was clearly demonstrated through scanning electron microscopy (SEM) analysis (Figure 2B). Compared with the unmodified pristine hydrogel region (AG), the hydrogel regions containing PIL (PG) and the composite containing PIL and NIPAM (PGN) exhibited markedly reduced internal pore sizes within their polymeric networks, with PGN displaying the most compact structure. This reduction in pore size directly reflects the intensified intermolecular interactions between polymer chains, primarily stemming from the synergistic effect of ionic interactions introduced by PIL and thermoresponsive hydrophobic aggregation triggered by NIPAM.

Next, we verified the conductivity of these hydrogels (Figure 2C and D). PGN showed the highest conductivity at 0.664 mS cm^−1^, close to the conductivity of human skin (2.6 × 10 ^−4^ mS cm^−1^), compared with previously reported electroactive hydrogels for wound repair, the PGN hydrogel developed in this study has more comprehensive advantages in physicochemical properties [53]. PG showed higher conductivity than AG, which exhibited a negligible conductivity of 0.043 mS cm^−1^. The presence of cationic groups in PIL considerably improved the conductivity of the hydrogels. To evaluate the effect of temperature variations on hydrogel conductivity, we measured the conductivity of AG, PG and PGN hydrogels at 25, 37 (Supplementary Figure S4) and 40°C (Supplementary Figure S5). The results showed that the conductivity of all hydrogels increased slightly with increasing temperature: the conductivity of PGN hydrogel was 0.664 mS/cm at 25°C, 0.712 mS/cm at 37°C and 0.738 mS/cm at 40°C, with an increase of only 7.2% and 11.1%, respectively, compared with that at 25°C. This mild change in conductivity is attributed to the enhanced ion migration rate in the hydrogel network with increasing temperature. More importantly, the conductivity of PGN hydrogel remained stable in the range of 25–40°C and was always close to the conductivity of human skin, which ensures that the hydrogel can stably output the directional electric field under physiological temperature and even mild fever conditions, without significant fluctuation of electrical properties caused by temperature changes.

Owing to the addition of NIPAM in PGN, we also compared the thermal responsiveness of PG and PGN at 37°C by periodically photographing them and recording the changes in their diameters (Supplementary Figure S6). After 80 min, the area of PG remained unchanged, whereas that of PGN contracted to 52.7% of the original area. This thermal response behavior can be attributed to the doping of NIPAM in the internal crosslinking structure of PGN, which changes the internal network structure and causes the structure to shrink with increasing temperature [54]. Therefore, when applied to skin at a temperature of 37°C, PGN/IC may help promote wound contraction and reduce wound tension, thereby accelerating wound healing.

The outer ring of the EH dressing was employed as the positive electrode, whereas the inner ring was employed as the negative electrode. Leveraging the exceptional mechanical stability and adhesiveness of PGN and AG, this configuration successfully integrates EH and ES components in the PGN/IC system (Supplementary Video S2). Power supply is achieved by transmitting the NFC signal from a mobile phone to the receiving conductive coil in PGN/IC. This design enables short-distance, contactless power transfer. The NFC functionality within the phone can be conveniently activated at any time and location. When NFC is activated, the current travels along the conductive coil of PGN/IC, passes through the circular skin interface electrode and enters the wound from its edge, establishing a transverse EF toward the wound (Supplementary Video S3).

To protect sensitive and fragile wounds, a dressing must exhibit appropriate adhesion, enable on-demand painless removal and possess good mechanical stability. Further investigation into the effect of temperature on dressing adhesion revealed that lowering the temperature to 4°C is key to regulating the adhesion behavior of PGN (Figure 2E and F). At room temperature (25°C), PGN could easily adhere to various surfaces, including steel, glass, rubber, polypropylene, polyethylene and skin. When cooled with an ice pack (4°C), PGN detached easily without leaving a residue. This notable adhesion behavior of PGN may be attributed to electrostatic interactions between polymer chains and the object interface, hydrogen bonding among chemical functional groups and residual interfacial forces contributed by remaining residual groups. The temperature reduction (4°C) changed the helical conformation of Gela-NH_2_, which greatly reduced adhesion to the interface, achieving painless removal of Figure 2F presents the tensile properties of AG, PG and PGN hydrogels.

Figure 2G and Supplementary Figures S7 and S8 presents the tensile properties of AG, PG and PGN hydrogels. The hydrogels exhibited strains exceeding 300%, with PGN exhibiting the highest strain at 378% and a strength of 153 kPa. Figure 2H and Supplementary Figure S9 presents the compression properties of the hydrogels. The compressive strain reached 85%, and the elastic moduli for AG, PG and PGN were 11 ± 2, 37 ± 6 and 51 ± 9 kPa, respectively. PG exhibited greater stiffness than AG owing to the addition of PIL, and PGN exhibited higher crosslinking density compared with PG owing to the addition of NIPAM, which may also be the reason for its enhanced mechanical stability. The rheological properties of the hydrogels were characterized by dynamic rheological tests. At both 25 and 37°C, the storage modulus (G′) of all hydrogels was consistently higher than the loss modulus (G″) (Figure 2I and J), indicating typical elastic gel behavior. The G′ values followed the order PGN > PG > AG, consistent with the tensile and compressive results, confirming that PIL and NIPAM improved crosslinking density and mechanical stability. Compared with 25°C, the G′ of PGN increased by 28% at 37°C, attributed to the thermosensitive hydrophobic aggregation of NIPAM. Temperature sweep tests (Figure 2K) showed that G′ of PGN remained stable and always higher than G″ in the range of 25–40°C, indicating stable gel state under physiological and mild fever conditions, which is beneficial for long-term wound application.

The water content of the three hydrogels ranged from 83% to 88% (Supplementary Figure S10). The swelling rate of AG exceeded 485% and that of PGN was the highest at 564% (Supplementary Figure S11). In addition, the hydrogels showed good hydrophilicity, with contact angles ranging from 28° to 37° (Figure 2L). PGN from the wound. The above characterization results confirm that the hierarchical structural design and component synergy of PGN/IC hydrogel endow it with ideal physicochemical and functional properties.

### Cell compatibility of hydrogels

**Figure 3:**
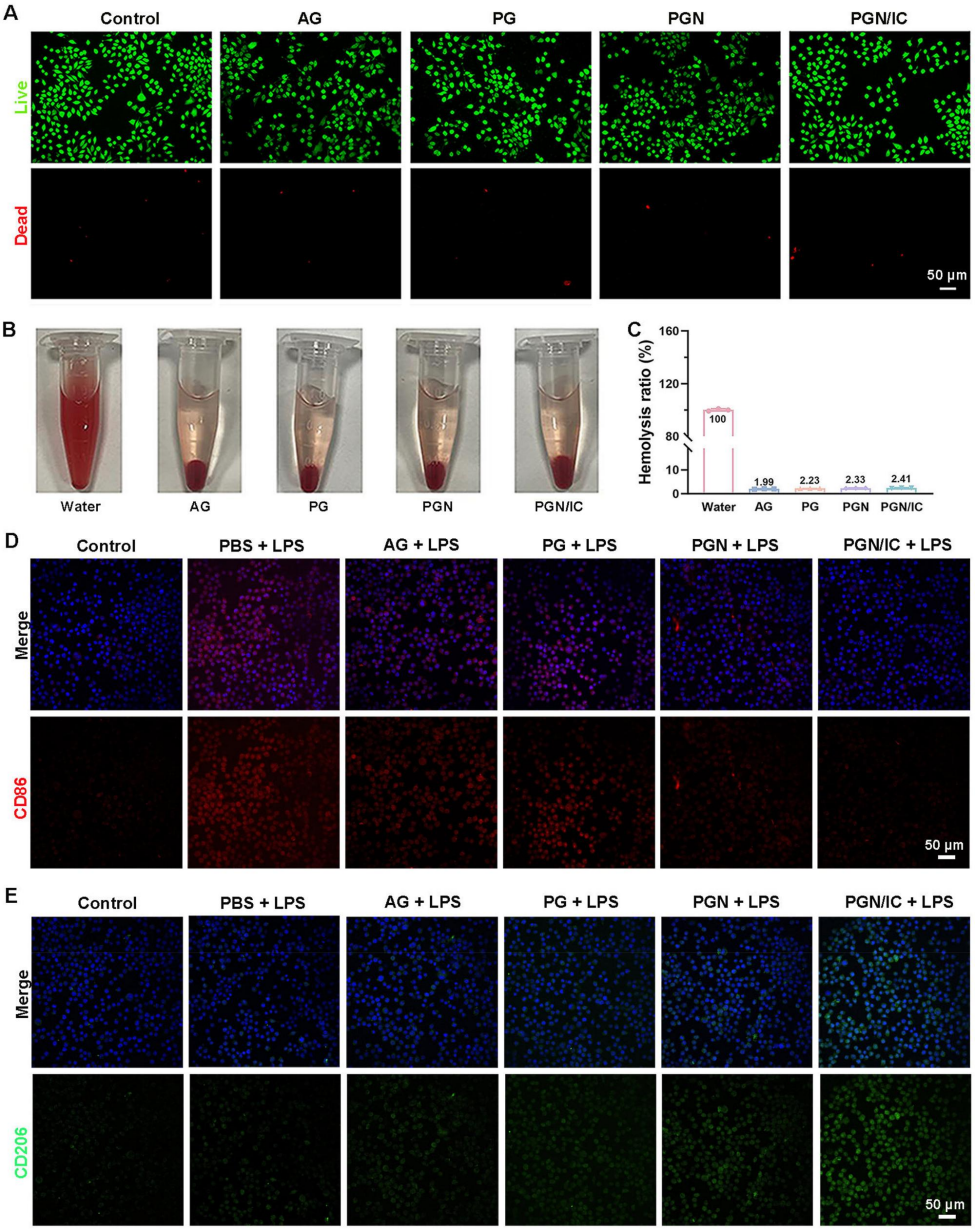
Biological safety and macrophage-regulating properties of PGN/IC. (**A**) Live/dead cell staining of L929 fibroblasts cultured for 24 h in hydrogel extract, with red representing dead cells and green fluorescence representing live cells. Scale bar: 50 μm. (**B, C**) Blood compatibility and statistics of different hydrogels. Merged confocal images in macrophages: (**D**) CD86 (red)/nucleus (blue) and (**E**) CD206 (green)/nucleus (blue), scale bar: 50 μm. *n* = 3 independent samples, data represent mean ± SD; *P < 0.05, **P < 0.01, ****P < 0.0001.

To assess the effects of the hydrogels on cell viability, L929 cells were incubated with hydrogel extracts and subsequently subjected to Cell Counting Kit-8 cytotoxicity assays (Supplementary Figure S12) and live/dead cell staining (Figure 3A). Unlike for the control group, only a few cells in the experimental groups exhibited red fluorescence after 24 h, with cell viability remaining approximately 90% after 3 d. To determine the adsorption capacity of the hydrogels, bovine serum albumin was used instead of cell waste for simulating wound exudate. All three hydrogels had an adsorption capacity of approximately 256 mg g^−1^ (Supplementary Figure S13). These results indicate that the hydrogels can absorb wound exudate, maintain wound moisture and create favorable conditions for wound healing. Blood compatibility was assessed by determining the hemolysis rates of the hydrogels (Figure 3B and 3C). All hydrogels exhibited hemolysis rates of <2.41%, in line with the national safety standard of <5%. Furthermore, the hemostatic efficacy of PGN significantly surpassed that of the control group (Supplementary Figure S14). This superior performance can be attributed to the dense polymeric network of PGN rapidly absorbing fluid from the blood, resulting in the concentration of platelets and coagulation factors, which accelerates the local coagulation cascade. Concurrently, the contractile force generated by hydrophobic aggregation physically compresses the vascular wound site, thereby effectively reducing the wound size. The exposed hydrophobic microdomains facilitate the adsorption of plasma proteins, such as fibrinogen, providing anchoring sites for platelet adhesion and effectively sealing damaged vessels, particularly capillaries experiencing oozing hemorrhage. This multifaceted mechanism led to a notable reduction in blood loss, as quantitatively demonstrated in Supplementary Figures S15, where the PGN group exhibited markedly lower blood loss compared with the control.

The *in vivo* biocompatibility of the wireless-powered hydrogel was evaluated by staining the major organs of diabetic mice with hematoxylin and eosin after 16 d of treatment (Supplementary Figure S15). First, the structures of the organs (lung, kidney, spleen, liver and heart) were confirmed to be normal, with no pathological manifestations such as inflammation, necrosis, or fibrosis. Second, compared with other treatment groups, the alveolar structures were intact in the hydrogel-treated group, without inflammatory cell infiltration or interstitial thickening. No abnormalities were found in the structures of the glomeruli and renal tubules in the kidneys. The hepatic lobule structure was intact, and no fatty degeneration or necrotic foci were observed. The myocardial fibers should be closely arranged, and no experimental group showed fiber rupture or inflammatory cell infiltration. In conclusion, our results indicated that PG, PGN and PGN/IC are relatively safe for diabetic mice over a treatment duration of 16 d.

### *In vitro* regulation of macrophage polarization

The “balance” of the immune microenvironment is crucial for tissue repair and inflammation regulation. Among immune cells, M1 macrophages (CD86) exhibit pro-inflammatory and immune-activating functions, while M2 macrophages (CD206) execute anti-inflammatory and tissue-repairing functions. We investigated the effects of PGN/IC hydrogels on macrophage phenotypes. Immunofluorescence staining clearly demonstrated that under lipopolysaccharide (LPS) stimulation, the red fluorescence (CD86) in the PGN/IC + LPS group was significantly weaker than that in the other treatment groups (Figure 3D and E;  Supplementary Figures S16 and S17); additionally, the green fluorescence (CD206) in the PGN/IC + LPS group was markedly stronger than that in other groups. The PGN + LPS group significantly promoted macrophage polarization from the pro-inflammatory M1 phenotype toward the anti-inflammatory M2 phenotype, which was mainly attributed to the synergistic effects of the key components in the PGN hydrogel, namely PIL, NIPAM and the bioactive gelatin matrix. Under LPS‑induced inflammatory conditions, PGN/IC treatment not only remarkably downregulated the expression of the M1‑related pro‑inflammatory marker CD86, but also significantly upregulated the expression of the M2‑associated anti‑inflammatory and tissue‑repair marker CD206, as compared with the AG, PG and PGN groups.

### Directional migration of HaCaT *in vitro*

Inductive coupling enables controlled transmission of electric energy from a mobile phone to the conductive coil in PGN/IC over short distances wirelessly [55]. The resonance effect of the conducting coil was exploited to avoid using wired electrodes. A conducting coil with a diameter of 2 cm was used. The end receiving the wireless power supply simulates the stable output of a mobile phone. As the output voltage of the receiving end increases, the voltage received and output by the transmitting end also increases, achieving 100% wireless transmission of current. The stability of the transmitting end plateaus after it reaches the maximum voltage (5 V; Supplementary Figure S18). On the basis of coil resonance, the maximum wireless transmission distance between the transmitting and receiving ends was determined to be 7 mm (Supplementary Figure S19). This distance may be related to the density and area of the conductive coils on both ends and can be customized according to specific application requirements.

**Figure 4:**
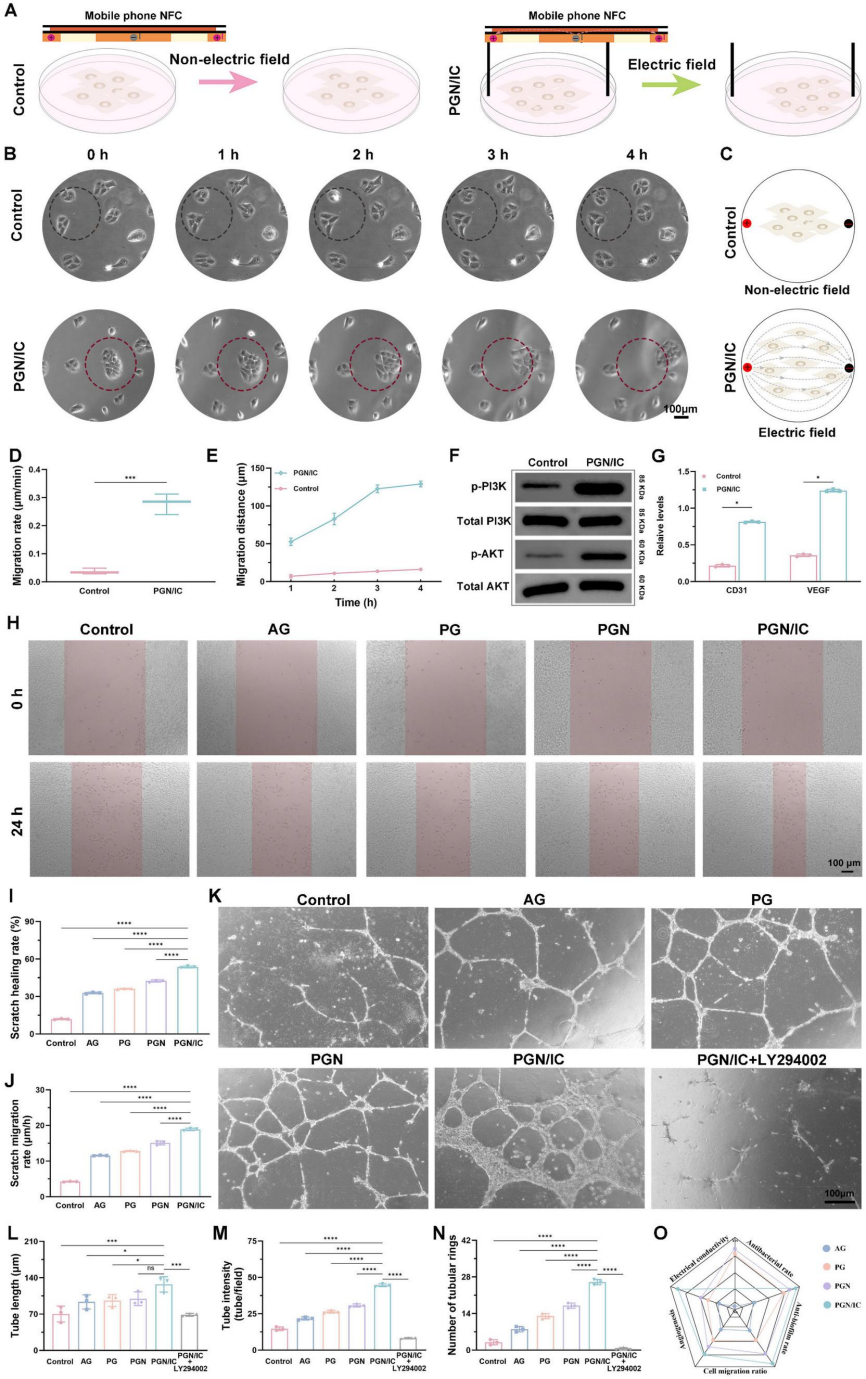
*In vitro* promotion of cell migration, proliferation and tube formation by PGN/IC. (**A**) Schematic diagram of cellular energization by PGN/IC. (**B**) Effect of wireless powered hydrogel PGN/IC on cell migration *in vitro*. Scale bar: 100 μm. (**C**) Schematic diagram of wireless powered hydrogel PGN/IC supplementing bioelectric field. (**D, E**) Cell migration rate and migration distance. (**F, G**) Western blot analysis of p-PI3K and p-AKT protein expression and gray value statistics in blank group and PGN/IC group. (**H–J**) Scratch experiment and scratch spacing statistics for L929. Scale bar: 100 μm. (**K–N**) Test tube formation and statistics of HUEVC cells after 24 h incubation with different hydrogels. Scale bar: 100 μm. (**O**) Omparative radar plot of key functional properties of different hydrogel dressings. *n* = 3 independent samples, data represent mean ± SD; *P < 0.05, **P < 0.01, ***P < 0.001, ****P < 0.0001, ns no significance.

To explore the effect of PGN/IC on HaCaT cells *in vitro*, we inoculated HaCaT cells in a modified petri dish to enable the observation of cell migration. The experimental group used the NFC wireless power transmission technology, allowing PGN/IC to apply an EF to the cells (Figure 4A), the magnitude of which was measured to be 120 mV mm^−1^ in the medium (Supplementary Figure S20). Live-cell workstation images (Figure 4B) showed that HaCaT cells in the control group exhibited minimal positional changes over 4 h, indicating weak, non-directional migration. Conversely, HaCaT cells in the PGN/IC group demonstrated significant directional migration over 2 h and migration out of the ring after 4 h (Supplementary Video S4). The motion trajectories of both cell groups are illustrated in Figure 4C, with the PGN/IC group exhibiting a 7-fold higher migration rate (Figure 4D) and covering 6.5-fold higher distance (Figure 4E) compared with the control. Cells in the PGN/IC group directionally migrated toward the negative electrode, which is aligned with the negative electrode of the wound center‘s endogenous EFs, promoting wound healing. Western blot analysis revealed significant upregulation of p-PI3K expression in the PGN/IC group, leading to increased expression of p-AKT downstream in the signaling cascade (Figure 4F and G). The PI3K/AKT pathway is critical for the proliferation and migration of epidermal cells. These results indicate that the EF applied by inductively coupled PGN/IC activates the PI3K/AKT pathway in HaCaT cells, protein expression levels were 2–3 times higher than those in the control group. Efficiently guiding the directional migration of epidermal cells toward the negative electrode. In conclusion, the EF output by inductively coupled PGN/IC activates the PI3K/AKT pathway in epidermal cells, regulating their electrotaxis and promoting high-speed migration toward the wound center, thereby facilitating wound healing.

The radar chart in Figure 4O summarizes the key functional indicators (electrical conductivity, antibacterial rate, anti-biofilm rate, cell migration ratio and angiogenesis) of AG, PG, PGN and PGN/IC hydrogels. AG showed the lowest performance across all indicators. PG exhibited improved antibacterial and anti-biofilm properties compared with AG, while PGN further enhanced conductivity and cell migration ability. PGN/IC achieved the highest values in all five dimensions, demonstrating its superior comprehensive performance in multiple wound repair-related functions.

### *In vitro* scratch migration and tube formation assay

Before examining the effects of PGN/IC on healing of infected wounds, we studied its influence on cell behavior *in vitro*. First, the effect of PGN/IC on proliferation of L929 cells was assessed using the scratch assay. The results showed that the distance between scratches was lower in the hydrogel-treated groups compared with the control group, implying that the hydrogels promoted L929 cell proliferation (Figure 4H–J). PGN/IC treatment elicited the highest scratch mobility at 53%, whereas the scratch mobility elicited by treatment with AG, PG and PGN ranged from 32% to 43%. This suggests that the inductive coupling–based EF applied by PGN/IC stimulates L929 cell proliferation and migration. Next, we conducted *in vitro* tube formation experiments using human umbilical vein endothelial cells (HUVECs) (Figure 4K–N), which demonstrated that PGN/IC most significantly promoted angiogenesis. The tube strength and length obtained after treatment with AG, PG and PGN were significantly lower than the corresponding values obtained after treatment with PGN/IC (tube strength: 15–30 vs. 45; tube length: 73–98 μm vs. 127 μm, respectively). Considering that EFs can affect angiogenesis, we hypothesized that PGN/IC might modulate the PI3K/AKT pathway in HUVECs. To test this hypothesis, the PI3K pathway inhibitor LY294002 was added to the PGN/IC + LY294002 group. PI3K, an intracellular phosphatidylinositol kinase and AKT, a serine/threonine-specific protein kinase, regulate protein synthesis, growth, proliferation and migration in many cell types and can be activated by various growth factors. The results suggested that the inductively coupled EF generated by PGN/IC potentially accelerates angiogenesis relative to the control and other treatment groups by activating the PI3K/AKT pathway in HUVECs.

### *In vitro* antimicrobial performance

**Figure 5:**
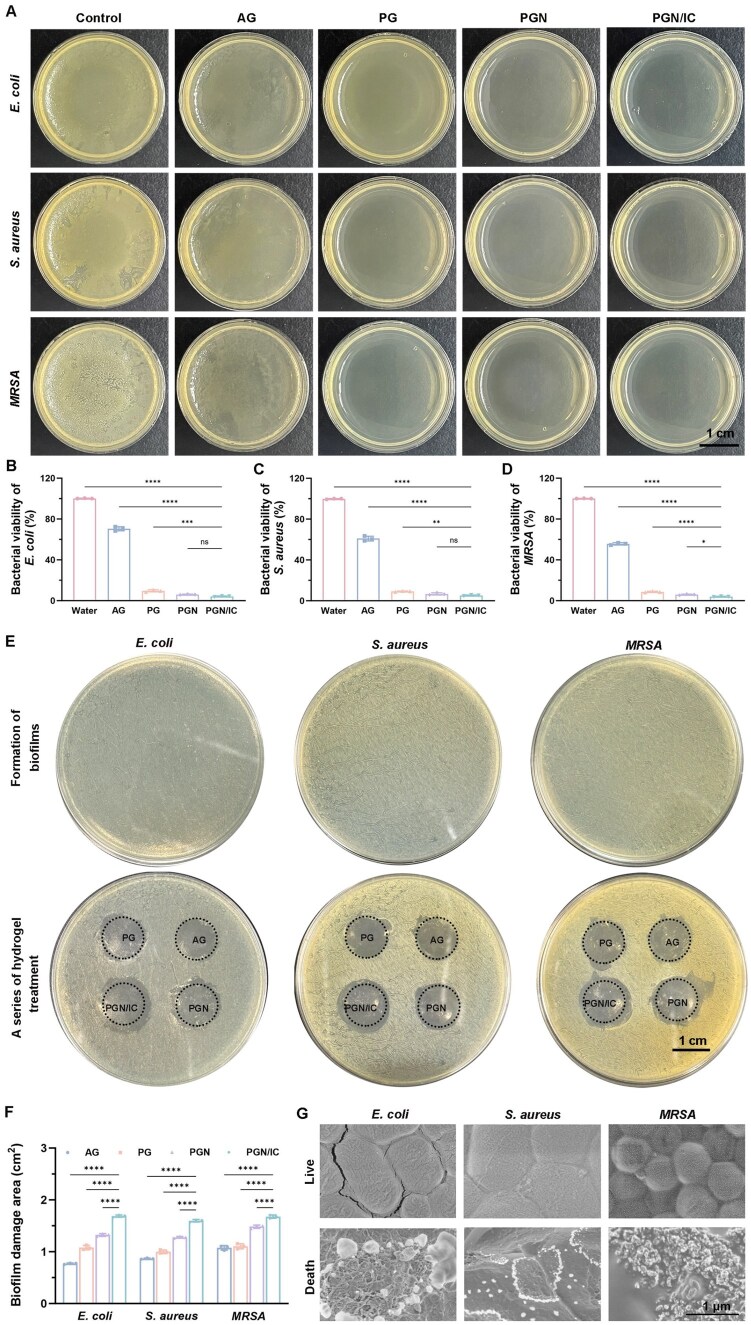
*In vitro* antibacterial activity evaluation of PGN/IC. (**A–D**) Quantitative evaluation of antimicrobial activity of different hydrogels. (**E, F**) The destructive ability of different hydrogels to biofilms produced by bacteria. Scale bar: 1 cm. (**G**) SEM images of bacteria before and after culture with hydrogel. Scale bar: 1 μm. *n* = 3 independent samples, data represent mean ± SD; *P < 0.05, ***P < 0.001, ****P < 0.0001.

Bacterial infection is one of the main reasons why the healing of diabetic wounds is hindered [56, 57]. Therefore, we tested the antibacterial efficacy of these hydrogels by incubating them with *Escherichia coli*, *Staphylococcus aureus* and methicillin-resistant *S. aureus* (MRSA) in liquid medium for 24 h (Figure 5A). The PG, PGN and PGN/IC groups demonstrated excellent antibacterial activity, with almost no colonies observed on solid agarose medium. Colony counting showed that PG, PGN and PGN/IC exhibited at least 84% antibacterial efficacy against *E. coli*, *S. aureus* and MRSA (Figure 5B–D), and this activity can be attributed to the cationic groups on PIL [58, 59]. Bacterial infections often involve the formation of bacterial biofilms. Bacteria can attach to the wound site within minutes, forming stronger adhesions over the next 4 h [60]. Over the next 8 h, they secrete proteins, polysaccharides, extracellular DNA and lipids, all of which contribute to the formation of a biofilm that inhibits drug treatment [61, 62]. To assess the inhibitory effects of AG, PG, PGN and PGN/IC on bacterial biofilms, *E. coli*, *S. aureus* and MRSA were cultured on plates for 12 h to allow biofilm formation, followed by treatment with hydrogels for another 12 h. The results showed that the hydrogels could disrupt the biofilm structure (Figure 5E and F). PGN/IC showcased the strongest biofilm-destroying ability and the most potent antibacterial activity, particularly against MRSA. The observed antibacterial efficacy of the hydrogels can be attributed to their intrinsic cationic antimicrobial properties, which cause efficient lysis of the biofilms.

After conducting the disk diffusion antibacterial assay, the bacteria inside and outside the disk were observed by SEM (Figure 5G). The bacteria outside the bacteriostasis zone were compactly arranged and full in shape, whereas those within the zone were fragmented and shapeless. Compared with similar electroactive hydrogels that rely on antibiotic loading or metal nanoparticles for antibacterial activity, the PGN/IC hydrogel developed in this study relies on the intrinsic antibacterial activity of PIL, which avoids the risk of bacterial resistance caused by antibiotics and the potential cytotoxicity of metal nanoparticles.

### *In vivo* healing of infected diabetic wounds

**Figure 6:**
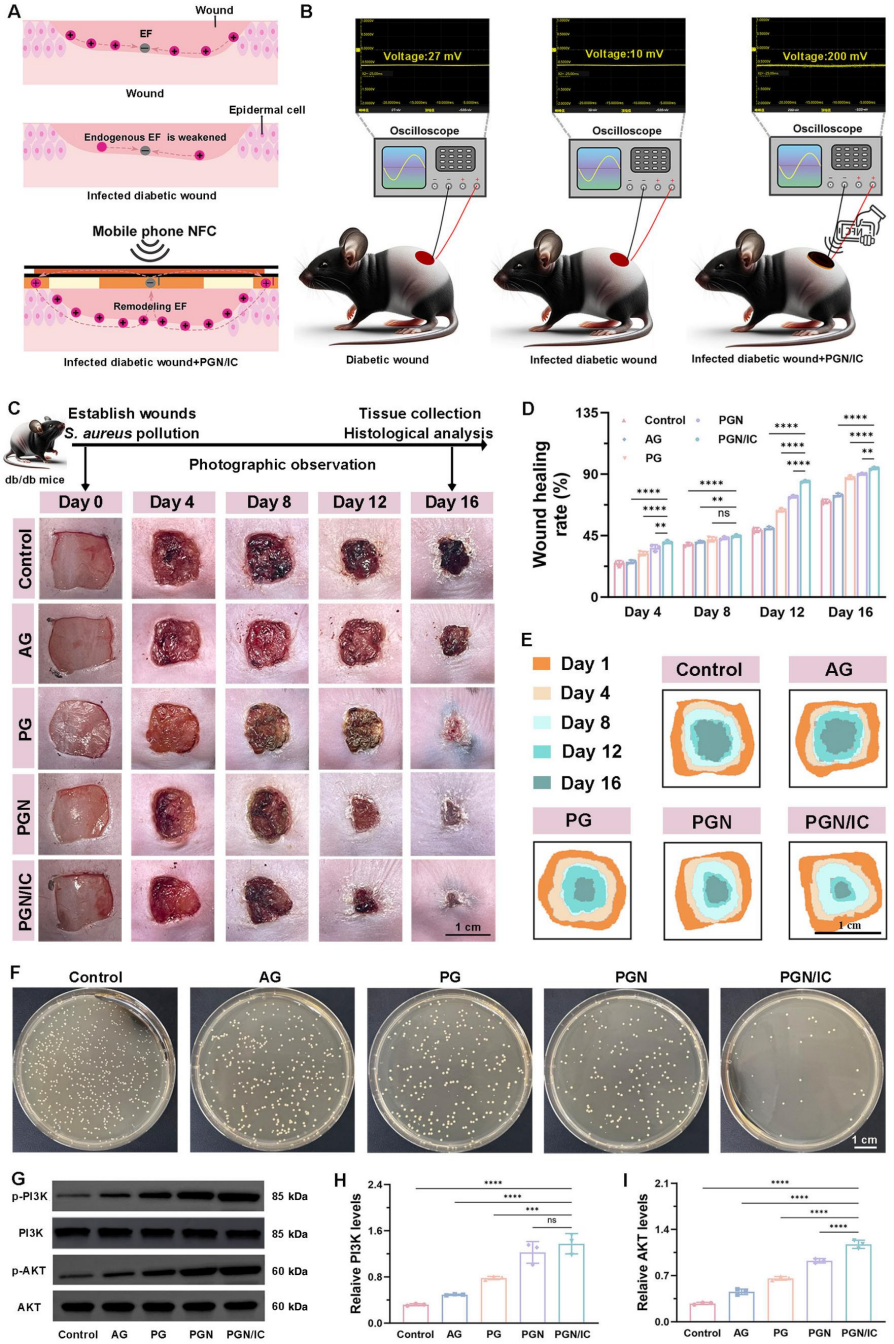
PGN/IC generates electrical stimulation precisely aligned with the EF via NFC, thereby promoting diabetic wound repair. (**A**) Schematic diagram of EFs in the trauma surface. (**B**) Oscilloscopemeasurements to record voltage schematics of diabetic mouse wounds, infected diabetic mouse wounds and PGN/IC-treated diabetic mouse wounds. (**C–E**) Photos, statistics and wound profiles of db/db mice infected by different treatment groups. Scale bar: 1 cm. (**F**) CFU of bacteria from wound beds on Day 4. Scale bar: 1 cm. (**G–I**) Western blot analysis of p-PI3K and p-AKT protein expression in different treatment groups. *n* = 3 independent samples, data represent mean ± SD; **P < 0.01, ***P < 0.001, ****P < 0.0001, ns no significance.

Our *in vitro* experiments demonstrated the beneficial effects exerted by PGN/IC on L929 cell proliferation, HUVEC angiogenesis and HaCaT cell migration. Next, we examined the therapeutic effect of PGN/IC on bacteria-infected diabetic wounds. The EF associated with acute wounds facilitates centripetal cell migration to promote tissue repair. However, in infected diabetic wounds, the EF is severely compromised due to a vicious cycle of persistent inflammation and bacterial infection within the microenvironment. To address this issue, we employed NFC stimulation to supplement the wound with a directed EF that is oriented toward the wound center in the form of the PGN/IC hydrogel system. This system exerts antibacterial effects while effectively restoring the wound EF, activating endogenous healing mechanisms to break the vicious cycle and accelerate the healing of infected diabetic wounds (Figure 6A).

A full thick-skin wound model (a 10 mm × 10 mm defect, 1 mm deep) was established on the back of spontaneous type 2 diabetic mice (db/db mice). A 200-μL bacterial suspension of *S. aureus* (1 × 10^8^ CFU/mL; CFU: colony-forming unit) was inoculated into the wound and allowed to be absorbed for 30 min, after which the wound was treated with different materials. The wound surface voltage was measured with an oscilloscope (Figure 6B). Bioelectric field reconstruction was found to break the inflammation-infection cycle. The baseline endogenous EF in murine diabetic wounds was 27 mV. Upon bacterial infection, this bioelectrical field was severely suppressed, dropping in intensity to 10 mV due to microbial disruption of transepithelial potential. Remarkably, the application of PGN/IC amplified the EF by 7.4-folds relative to the endogenous value to 200 mV through NFC-mediated wireless stimulation. This engineered EF amplification directly countered infection-induced EF suppression by repolarizing the wound margins, breaking the pathological inflammation cycle through EF-dependent downregulation of pro-inflammatory cytokines and accelerating re-epithelialization by enhancing directional keratinocyte migration. The 200-mV field intensity exceeds physiological thresholds, creating an electroceutical microenvironment that actively coordinates healing cascades beyond passive barrier functions.

To assess the effect of wireless power on wound healing, the therapeutic efficacies of individual dressings were compared to that of the wireless-powered PGN/IC system. During the bacteria-induced inflammatory phase (Day 4), the healing rate in the PGN/IC group was 35%, slightly lower than that in the PGN group (37%) but substantially higher than that in the control group (25%; Figure 6C and D). By Day 12, wounds in the PGN/IC group were approximately 85% healed, which was significantly greater than that observed in any other group. By Day 16, wound healing in the PGN/IC group was nearly complete (95%), compared with 90%, 88%, 75% and 70% in the PGN, PG, AG and control groups, respectively. Superimposing the wound areas from all time points provides an intuitive comparison (Figure 6E). The ability of wound dressings to resist microbial colonization is critical for mitigating infection risks during healing. To quantitatively evaluate the antimicrobial efficacy of PIL-based hydrogels, we performed standardized CFU assays against *S. aureus*, a prevalent multidrug-resistant wound pathogen. The PGN/IC composite demonstrated the most potent inhibition, lowering *S. aureus* viability by >99% within 4 d (Figure 6F). This rapid biocidal activity stems from three mechanisms, Cationic surface charge amplification: Quaternary ammonium groups in PIL electrostatically disrupt bacterial membranes. Synergistic contact killing: Thermoresponsive contraction due to NIPAM enhances physical contact with pathogens. Biofilm penetration: The dense yet hydratable network facilitates diffusion of bactericidal ions.

Additionally, we explored the effect of PGN/IC on the PI3K/AKT pathway in infected wound skin (Figure 6G–I). Western blotting results indicate that endogenous factors produced by PGN/IC significantly upregulate the expression levels of proteins in the PI3K/AKT pathway. Higher infection wound healing rates correlate with increased expression levels of PI3K and AKT. These findings suggest that the EF supplemented by PGN/IC activates the PI3K/AKT pathway, guiding cells with electrotaxis to undergo directed migration, promoting cell proliferation and accelerating angiogenesis. In summary, PGN as an EH dressing exhibits good antibacterial properties, efficiently neutralizes drug-resistant bacteria, alleviates wound bacterial infection, absorbs metabolic waste and creates a moist microenvironment in the wound. PGN also serves as a skin interface electrode, supplementing endogenous EFs to wounds on demand via mobile phone NFC stimulation based on the principles of inductive coupling. This efficiently guides epidermal cell migration, promotes cell proliferation and angiogenesis and enhances the treatment of infected wounds.

### Histological evaluation of wound healing

**Figure 7:**
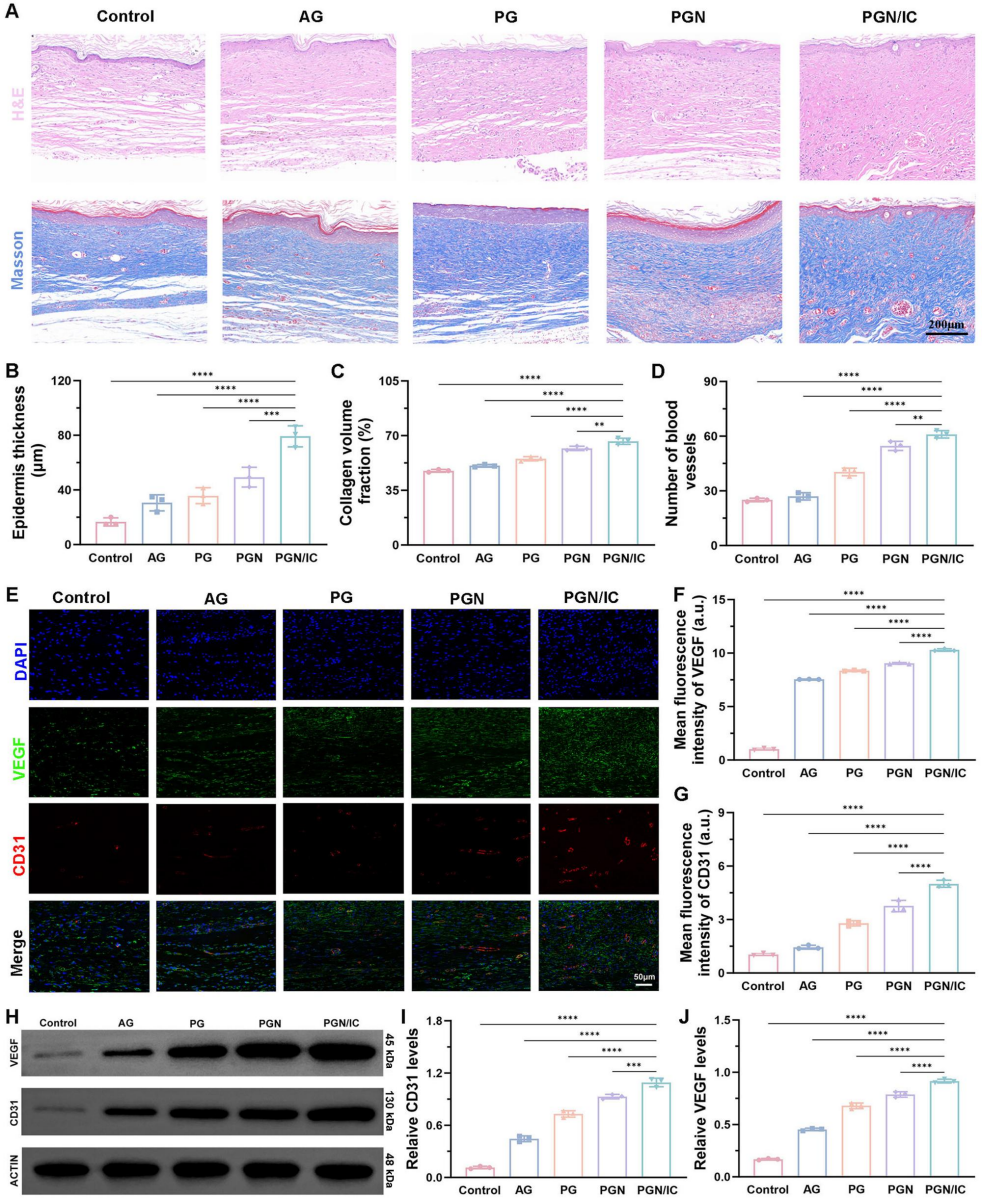
Histopathological assessment of infected wound healing in different treatment groups. (**A–D**) H&E staining and statistics on the 16th day of wound healing: collagen volume fraction, number of blood vessels and epidermal thickness. Scale bar: 200 μm. (**E–G**) The immunofluorescence staining of VEGF (green) and CD31 (red) and the relative area coverage were measured 16 days after wound healing in each group. Scale bar: 50 μm. (**H–J**) Western blot was used to detect the expression of VEGF and CD31 in different treatment groups. *n* = 3 independent samples, data represent mean ± SD; **P < 0.01,***P < 0.001, ****P < 0.0001.

We studied the effects of different dressings on the re-epithelialization of the infected wound in db/db diabetic mice. Moreover, the vascular and epidermal thickness of collagen I and collagen III in the wound regeneration tissue was evaluated. After 16 d, the regenerated tissue of the infected wound was subjected to hematoxylin and eosin staining and Masson staining (Figure 7A). The average epidermal thickness of regenerated tissue in the PGN/IC group was 78 μm, significantly greater than the thicknesses of 51 and 17 μm in the PGN and control groups, respectively (Figure 7B). We found that collagen I and collagen III deposition in the PGN/IC group was 66%, significantly higher than the depositions of 59%, 54%, 51% and 47% in the PGN, PG, AG and control groups, respectively (Figure 7C). Regarding angiogenesis, the PGN/IC group and PGN group exhibited 60 and 54 blood vessels per unit area, respectively, whereas the control group had at least 25 blood vessels per unit area (Figure 7D). This trend was even more pronounced in case of epidermal thickness. Although PGN alone promoted the healing of infected wounds owing to its high antibacterial efficacy, the wireless-powered PGN/IC significantly improved treatment speed and effectiveness. These results indicate that the inductively coupled PGN/IC not only retains the advantages of EH dressings but also efficiently reconstructs the endogenous EFs on the wound surface, activates the PI3K/AKT pathway, guides the directional migration of epidermal cells and accelerates wound closure. We also performed double immunofluorescence staining for vascular endothelial growth factor (VEGF) and platelet–endothelial cell adhesion molecule (CD31; Figure 7E). The mean percentage of VEGF staining in the PGN/IC group (1023%) was significantly higher than that in the PGN (899%), PG (832%) and AG groups (754%; Figure 7F).

Similarly, the PGN/IC group displayed the highest average percentage of CD31 staining (493%), whereas the AG group displayed the lowest (137%; Figure 7G). These results were corroborated by Western blotting findings, which also demonstrated that VEGF and CD31 expression levels were significantly higher in the PGN/IC group compared with the other groups (Figure 7H–J). The trends observed in immunofluorescence staining for growth factors were consistent across the groups. This suggests that the inductively coupled PGN/IC system not only reconstructs endogenous EFs but also stimulates the expression of growth factors in the wound tissue, accelerating angiogenesis and cell proliferation. Together with the reconstructed EFs, these upregulated growth factors efficiently guide epidermal cell migration, achieving high-quality wound healing.

### Mechanisms promoting healing of infected diabetic wounds

**Figure 8:**
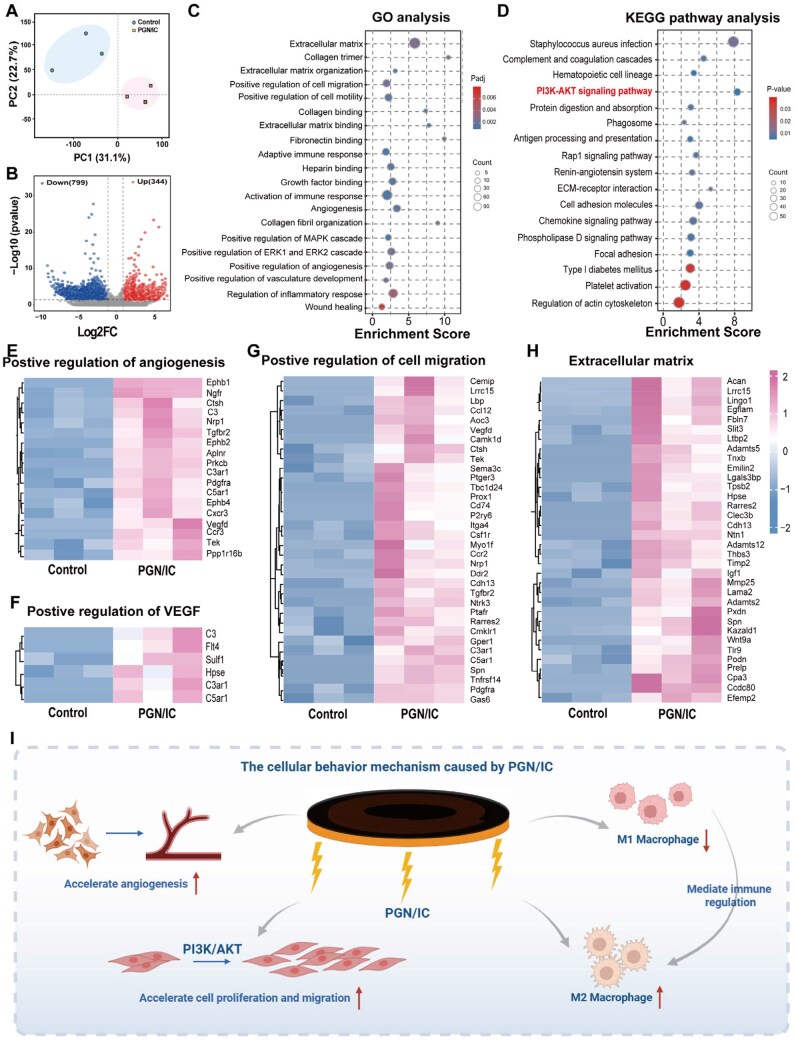
RNA sequencing analysis of wound regenerative tissue. (**A**) Principal component analysis (PCA) of differentially expressed genes in wound tissue from different groups of mice on Day 14. (**B**) Volcano plot of identified up- and down-regulated genes. (**C**) Gene ontology (GO) enrichment analysis of identified differentially expressed genes. (**D**) KEGG pathway enrichment analysis of identified differentially expressed genes. (**E, F**) Heatmaps of significantly upregulated angiogenesis-related and VEGF-related genes in the PGN/IC group (fold change ≥2 and *P* value adjusted (*P*_adj_) < 0.05). (**G, H**) Heatmaps of significantly upregulated genes involved in cell migration and extracellular matrix formation in the PGN/IC group (fold change ≥2 and *P*_adj_ < 0.05). (**I**) Regulatory mechanisms of PGN/IC behavior in diabetic wound infection.

To elucidate the mechanism through which wireless-powered PGN/IC promotes tissue regeneration, mouse tissues were collected on Day 14 for transcriptomic analysis. In unsupervised principal component analysis, the PGN/IC group exhibited significantly distinct gene expression patterns compared with the control group (Figure 8A). Volcano plots and Venn diagrams comparing the control and PGN/IC groups revealed significantly differentially expressed genes (Supplementary Figure S21), with 344 genes differentially upregulated in the PGN/IC group (Figure 8B). Gene Ontology enrichment analysis indicated that PGN/IC treatment positively regulated the extracellular signal-regulated kinase 1/2 cascade and significantly upregulated genes associated with wound healing processes, including angiogenesis, cell migration, collagen deposition and extracellular matrix formation (Figure 8C). Kyoto Encyclopedia of Genes and Genomes pathway analysis indicated that the PI3K-AKT signaling pathway, associated with activating the expression of heme oxygenase-1, was highly correlated with the wound healing mechanism of PGN/IC (Figure 8D). Notably, PGN/IC markedly upregulated angiogenesis-related genes (*Vegfd*, *Nrp1*, *Tgfbr2* and *Ephb4*) and VEGF-related genes (*Sulf1* and *C5ar1*; Figure 8E and F). Additionally, genes associated with cell migration (*Pdgfra*, *Tgfbr2* and *Prox1*), extracellular matrix formation, growth factor binding and suppressing IL-6 production were significantly upregulated following PGN/IC treatment (Figure 8G and H). Consistent with previous studies, both *in vitro* and *in vivo* experiments in this study demonstrated that PGN/IC, when applied to infected diabetic wounds, stimulated growth factor release and pathway activation, accelerates angiogenesis, promotes cell proliferation and migration and mediates macrophage reprogramming to achieve rapid and effective healing responses. By reconstructing the bioelectric field, this mechanism achieves timely macrophage polarization from pro-inflammatory to anti-inflammatory and reparative phenotypes—a transition that is critical for tissue healing (Figure 8I).

## Discussion

The construction of a directional biomimetic electric field is rooted in the inherent physiological characteristics of the endogenous electric field at the wound site, that is, the strict directionality extending from the wound periphery to the center, which serves as a critical electrical signal for driving cell migration [33, 63, 64]. Based on this, the design of a directional biomimetic electric field is not only highly consistent with the electric field microenvironment of the wound, but also an essential prerequisite for improving repair efficiency. Unlike the non-directional electric fields of electrical stimulation, the directional electric field generated by the PGN/IC system can precisely drive the directional electrotactic migration of epidermal cells, activate the PI3K/AKT pathway and mediate the remodeling of the immune microenvironment. Furthermore, this system can downregulate the expression of the pro-inflammatory marker CD86 and simultaneously upregulate the expression of the anti-inflammatory and pro-repair marker CD206, thereby driving the M2 phenotypic polarization of macrophages to alleviate excessive inflammation and establish a regenerative microenvironment conducive to tissue repair.

The stable and non-invasive output of the directional biomimetic electric field depends on an optimized power supply mode. Compared with conventional external wired power supplies, the NFC wireless power supply adopted in this study exhibits distinct advantages in the electrical stimulation repair of diabetic wounds [65]. Via near-field wireless induction, NFC achieves a wireless and physical interface-free power delivery mode, which can effectively avoid thermal injury and infection risks caused by wired power supplies [66, 67]. Furthermore, relying on wireless power supply, NFC enables precise cellular electrical regulation. Its miniaturized and flexible modules can be perfectly integrated with concentric circular-structured hydrogel electrodes to construct a PGN/IC system that combines wearing comfort and clinical practicability. This power supply mode overcomes the inherent drawbacks of traditional wired power supplies in terms of safety and integration, providing core technical support for the non-invasive, long-acting and precise electrical repair of diabetic wounds [68, 69].

The regulatory effect of the PGN/IC system on wound repair is essentially mediated by the activation of the PI3K/AKT signaling pathway, which serves as a core pathway regulating inflammatory response, cell motility and angiogenesis during wound repair and is crucial in diabetic chronic wounds [37]. Activation of this pathway inhibits excessive activation of NF-κB, downregulates pro-inflammatory factors and upregulates anti-inflammatory pro-repair markers (e.g. downregulating CD86 and upregulating CD206), thereby driving M2 polarization of macrophages to alleviate chronic inflammation and establish a favorable regenerative microenvironment [68, 70]. Meanwhile, it enhances the directional migration and proliferation of epidermal cells and fibroblasts, mediates endothelial cell tube formation and upregulates pro-angiogenic factors to improve the ischemic and hypoxic state of wounds [53]. In summary, the PGN/IC system achieves triple synergistic regulation of anti-inflammation, pro-cell migration and pro-angiogenesis by activating the PI3K/AKT pathway via directional biomimetic electric fields, precisely targeting the three core pathological defects of diabetic wounds.

In addition to the above electroregulatory and molecular pathway activation effects, the antibacterial property of the PGN/IC system, derived from PILs, further ensures the safety and effectiveness of long-term wound repair. In recent years, ionic liquids (ILs) have attracted extensive attention in the field of biomedical materials owing to their antibacterial activity, high ionic conductivity, excellent electrochemical stability and biocompatibility [71]. In this study, ILs were combined with poly(acrylic acid) (PAA) to synthesize PILs, which were then doped into the gelatin network in trace amounts to fabricate EHs. Relying on the inherent antibacterial properties of PILs, this strategy not only significantly improves the conductivity of the prepared hydrogels but also avoids the problem of bacterial resistance induced by conventional antibiotic additives, making it highly suitable for long-term safe and stable biomedical scenarios such as diabetic wound treatment [72].

To summarize, this study integrates near-field communication (NFC) wireless power supply, directional biomimetic electric fields, PI3K/AKT pathway activation and polyionic liquid (PIL)-based antibacterial properties into the PGN/IC system for the first time. This system not only addresses the limitations of traditional wound dressings (e.g. single function and limited efficacy) and wired electrical stimulation systems (e.g. inconvenient operation and high infection risk), but also achieves synergistic regulation of inflammation, cell migration, angiogenesis and infection control through a multi-level regulatory network. The design of the concentric circular EH is highly consistent with the physiological repair mechanism of wounds, providing solid experimental and theoretical support for the clinical translation of efficient, safe and non-invasive diabetic wound repair technologies.

Compared with current electroactive dressings for diabetic wound repair, this work demonstrates four major novelties. First, we integrate smartphone‐enabled NFC wireless power into electroactive hydrogels for the first time, constructing a battery‐free, wireless, noninvasive directional electrical stimulation system that overcomes the limitations of conventional wired and rechargeable devices. Second, the concentric circular sandwich‐structured hydrogel electrode generates a directional biomimetic electric field consistent with the endogenous wound electric field, enabling more efficient directional migration of reparative cells. Third, we establish a triple synergistic strategy involving bioelectric field reconstruction, broad‐spectrum antibacterial activity and immune regulation, which simultaneously targets the three core pathological barriers of infected diabetic wounds. Fourth, the PGN/IC dressing shows favorable mechanical adaptability, thermosensitive painless detachment and good biocompatibility, holding high potential for outpatient and home‐based chronic wound management. Nevertheless, this study has several limitations: the therapeutic efficacy and safety need further validation in large animal models; and the NFC electrical stimulation parameters should be optimized for different wound healing stages to further enhance therapeutic outcomes.

## Conclusion

This study successfully developed a wireless multifunctional EH dressing named PGN/IC, which targets the three core pathological barriers of infected diabetic wounds: disrupted bioelectric fields, bacterial infection and immune dysregulation. By integrating the concentric-circle sandwich hydrogel structure with IC and smartphone NFC wireless power supply, PGN/IC generates a precisely controllable 3D directional EF that mimics physiological wound bioelectrical signals, effectively reconstructing the directional EF of infected wounds to accelerate tissue repair.

The composite hydrogel exhibits favorable mechanical flexibility, temperature-responsive behavior and excellent biocompatibility. It possesses intrinsic antibacterial activity to disrupt drug-resistant bacterial biofilms and activates the PI3K/AKT pathway via directional EF to significantly promote epidermal cell migration/proliferation and angiogenesis. Additionally, PGN/IC remodels the immune microenvironment by downregulating the pro-inflammatory marker CD86 and upregulating the anti-inflammatory/repair marker CD206, restoring immune homeostasis in infected wounds.

Collectively, this work pioneers a synergistic strategy combining NFC wireless power and EH design to realize noninvasive, controllable directional ES, integrating bioelectric field reconstruction, broad-spectrum antibacterial activity and immune regulation. This multifunctional platform overcomes the drawbacks of traditional ES devices and provides a promising clinical solution for chronic infected diabetic wounds. Future work will focus on optimizing the efficiency of wireless energy transmission and electric field parameters, elucidating the synergistic mechanisms of multiple signaling pathways and advancing the preclinical validation and clinical translation of this platform. This strategy possesses great transformative potential for the targeted therapy and repair of complex tissue injuries.

## Supplementary Material

rbag115_Supplementary_Data
