# Supplementary material for: An electroactive platform enabled by near-field communication for accelerating infected diabetic wound healing via directional electric field reshaping and immunomodulation
Source: Regen Biomater. 2026 Jun 9;13:rbag115. doi: 10.1093/rb/rbag115 (PMC13346114; doi:10.1093/rb/rbag115)
Supplement: rbag115_Supplementary_Data [file rbag115_supplementary_data.zip › Supporting Information.docx]

**Supporting Information**

**An Electroactive Platform Enabled by Near-Field Communication for Accelerating Infected Diabetic Wound Healing via Directional Electric Field Reshaping and Immunomodulation**

Yuange Zong ^1^, Ying Chen ^1^, Kexin Deng ^1^, Peng Zheng ^1^, Hongling Zhou ^1^, Ze Zhang ^1^, Wanqi Huang ^1^, Danyang Huang ^1^,* Yuan Peng ^1^,* and Jiaping Zhang ^1^,*

^1^ Department of Plastic Surgery, State Key Laboratory of Trauma and Chemical Poisoning, Southwest Hospital, Army Medical University (Third Military Medical University), Chongqing 400038, China.

* Correspondence address. E-mail :

[danyanghuang@tmmu.edu.cn](mailto:danyanghuang@tmmu.edu.cn) (H.D.Y.);

[pengyuan9514@tmmu.edu.cn](mailto:pengyuan9514@tmmu.edu.cn) (Y.P.);

japzhang@tmmu.edu.cn(J.P.Z.)

**1 . Materials and Methodology**

**1.1 Synthesis of polyionic liquids**

The synthesis process of the ionic liquid is shown in Figure 2A. Specifically, 1-vinylimidazole (1 mmoL) and 4-bromomethylbenzaldehyde (1 mmoL) are first mixed in anhydrous acetonitrile (50 mL) and heated at 90℃ for 8 hours. Yellow ionic liquid 4- (1h-vinylimidazole) -1-methylene) benzoic acid was obtained by silica gel chromatography using dichloromethane/methanol (20/1, v/v) as eluent. Its synthesis has been confirmed in our previous article ^[1-2]^.

**1.2 Synthesis of amino gelatin**

The synthesis route of amino gelatin is shown in Figure 2B. In short, the carboxyl group in gelatin reacts with ethylenediamine to amino group with the help of DMTMM. Gelatine B (7.65 mmoL) was dissolved in 80 mL PBS (0.1 M), followed by the addition of ethylenediamine (249.58 mmoL), the pH of the solvent was adjusted to 6.5, and then DMTMM (6.78 mmoL) was added. The reaction lasted for 8 hours at room temperature. After 72 hours of deionized water dialysis, the amino gelatine could be obtained by freeze-drying.

**1.3 Scanning Electron Microscopy.**

The prepared hydrogel is freeze-dried by a vacuum freeze dryer (Xinyi-10A). The freeze-dried hydrogel is then cut open to reveal the internal cross-sectional aperture. Then it was attached to the copper platform with conductive adhesive and sprayed with gold. The microstructure of the sample was visually characterized by SEM.

**1.4 Measurement of electrical conductivity**

The conductivity of the hydrogels was measured by electrochemical impedance spectroscopy (EIS) at a constant temperature of 25 °C (room temperature), with a frequency range of 0.1 Hz to 10⁵ Hz and an amplitude of 10 mV. The conductivity (σ) was calculated by the formula: σ = L/(R×S), where L is the thickness of the hydrogel sample, R is the bulk resistance obtained from the EIS Nyquist plot, and S is the contact area of the sample with the electrode.

**1.5 Temperature sensitive test**

The PGP and PG hydrogels were made into cylinders with a diameter of 40 mm and a height of 3 mm, which were then placed in an incubator at 37°C and measured and photographed at specified times of 0, 20, 40, 60 and 80 minutes. The diameter of the hydrogel sample was measured by ImageJ software.

**1.6 Adhesion evaluation**

Using steel, glass, rubber, PP, PE and pig skin as substrates, the adhesion ability of hydrogel to substrates was tested. Simply say that the substrate is cut into a square (20 mm×20 mm) and kept clean before use. Bond strength was tested on a universal material testing machine (Instron 3344, USA) equipped with a 25 N load cell at a speed of 2 mm min^-1^.

**1.7 Mechanical property test**

The hydrogels were cut into cuboid (25 mm×10 mm×5 mm) and cylindrical (10 mm diameter, 10 mm height) shapes and subjected to tensile and compression tests via a material testing machine (Instron 3344, USA) equipped with a 25 N load cell. The speed is 10 mm min^-1^.

**1.8 Rheological Measurements**

Rheological measurements were performed using a rotational rheometer (Model Antonpaar, ) equipped with a parallel-plate geometry (20 mm diameter, 1 mm gap). All hydrogel samples were prepared to a consistent cylindrical shape (20 mm diameter, 2 mm height) and equilibrated at the testing temperature for 5 min prior to each measurement. Frequency sweep tests were conducted at 25 °C and 37 °C, over a frequency range of 1–100 rad/s, under a constant strain of 1% (within the linear viscoelastic region). Temperature sweep tests were performed from 25 °C to 40 °C at a heating rate of 1 °C/min, with a constant frequency of 1 Hz and a constant strain of 1%. The storage modulus (G′) and loss modulus (G″) were recorded as functions of frequency and temperature, respectively.

**1.9 Water content**

The prepared hydrogel was first weighed and recorded, and then weighed again and recorded the hydrogel after freeze-drying by a vacuum freeze dryer (Xinyi-10A). Water content =(Wo-W_A_)/W_O_×100%. Where W_A_ is the quality of hydrogel after freeze-drying, W_O_ is the quality of hydrogel before freeze-drying.

**1.10 Swelling rate**

Swelling tests were performed on PBS(0.1M pH=7.4) at 37°C. The initial weight of the hydrogel is called S_O_. Remove the hydrogel in the swelling process at different times, after the filter paper absorbs the water stains on the surface, record the weight of the hydrogel S_T_, swelling rate = (S_T_-S_O_)/S_O_×100%.

**1.11 Contact Angle test**

Made of hydrogel to prevent surface contact Angle instrument (Beidou Instrument, CA500S) platform, drop droplets, through the software to take photos to observe the size of the contact Angle.

**1.12 BSA adsorption evaluation**

BSA-FITC was used as a simulated protein. First, the hydrogel was cut into cuboids (10 mm×10 mm×20 mm), weighed and disinfected under ultraviolet for 30 minutes. Under the condition of avoiding light, the hydrogel was incubated with 10 mL BSA-FITC (10 mg mL^-1^) solution on a shaking table at 150 rpm at 37°C for 24 hours. The residue of BSA in the supernatant was then determined by ultraviolet spectrophotometer (Shimadzu UV-2550) at 595 nm. The adsorption rate of BSA =(B_0_-B_X_)×V/W. Where B_0_ is the absorbance before BSA incubation, B_X_ is the absorbance after BSA incubation, V is the volume added to BSA, and W is the mass of hydrogel.

**1.13 Hemolysis experiment**

For the PGN/IC group, the hydrogel was pre-assembled with the NFC induction coil module, and the NFC wireless power supply was activated by a smartphone for 10 min every 30 min during the 4 h incubation period with diluted mouse red blood cell suspension, with a stable output voltage of 1.0 V and electric field intensity of 120 mV/mm. The rest of the operation steps were consistent with the AG, PG, and PGN groups. Briefly, hydrogel samples were immersed in normal saline at 37 °C for 30 min, then added to the diluted red blood cell suspension and incubated at 37 °C for 4 h. After centrifugation, the absorbance of the supernatant at 540 nm was measured to calculate the hemolysis rate. Deionized water was used as the positive control. Hemolysis rate = (OD_G_-OD_P_)/(OD_W_-OD_P_)×100%. OD_G_ represents OD value of hydrogel, OD_W_ represents OD value of negative control, and OD_P_ represents OD value of positive control.

**1.14 In Vivo Biocompatibility Evaluation**​

To assess the systemic biocompatibility of the PGN/IC hydrogel, mice were euthanized at 16 days post-treatment, and major organs including heart, liver, spleen, lung, and kidney were promptly excised. Tissues were fixed in 4% paraformaldehyde for 24 h, followed by dehydration through a graded ethanol series, clearing in xylene, paraffin embedding, and sectioning at approximately 4 μm thickness. Sections were stained with hematoxylin and eosin (H&E) and examined under a light microscope for morphological alterations. Particular attention was paid to detecting inflammatory cell infiltration, architectural disruption, necrosis, or other pathological abnormalities. At least three animals per group were evaluated, and all histological assessments were performed independently by two pathologists in a blinded manner to ensure objectivity and reproducibility of the results.

**1.15 Cytotoxicity test**

L929 was used as the experimental model of cell biocompatibility. L929 cells (1 ×10^4^ cells /mL) were inoculated in 96-well culture plates for 24 hours, and then cultured in the medium soaked with different hydrogels. After 1 day of culture, L929 was stained for 30 minutes with Calcein/PI cell activity and cytotoxicity detection reagents, respectively, under the condition of dark light. The cell morphology is then observed and images are taken using a Living cell workstation (Leica DMI6000B). At the same time, the cell viability was determined by CCK-8 method after 1, 2 and 3 days of co-culture of the hydrogel-soaked medium with L929. The OD value of the mixture at 450 nm was measured by Tecan Infinite F50. Cell viability=(OD_G_-OD_C_)/(OD_O_-OD_C_)×100%, where OD_G_ is the OD value of the experimental group, OD_O_ is the OD value of the blank group, and OD_C_ is the OD value of 10% CCK-8 solution.

**1.16 Scratch test**

Using L929 as the cell model, cells (1×10^5^ cells mL^-1^) were inoculated in 6-well plate petri dish. When the cell density reached 85 %-90 %, marks were evenly marked on the well plate with sterile gun head. After washing with PBS, pictures were taken through microscope, and sterilized hydrogel (1 g) was then placed for coculture for 24 hours. The distance between the scratches was measured using the ImageJ software.

**1.17 In vitro tube forming test**

HUVEC was used as the experimental model of tube formation in vitro. HUVEC was incubated with hydrogel extract for 24 hours. The formation images of HUVEC cells were obtained under microscope, and the tube lengths of 4 random fields of view were analyzed by ImageJ software.

**1.18 Antibacterial performance evaluation**

For the PGN/IC group in the planktonic antibacterial assay, the hydrogel was co-incubated with E. coli, S. aureus, and MRSA bacterial suspensions (1×10⁶ CFU/mL) at 37 °C for 24 h. During the incubation period, the NFC wireless power supply was activated for 10 min every 30 min, with a stable output voltage of 1.0 V and electric field intensity of 120 mV/mm. The rest of the operation steps were consistent with the AG, PG, and PGN groups. After incubation, bacterial suspensions were gradient diluted and coated on agar plates for colony counting, and the antibacterial rate was calculated.

**1.19 Evaluation of antimicrobial biofilms**

For the biofilm destruction assay, pre-formed bacterial biofilms were co-incubated with PGN/IC hydrogel for 12 h, with the same NFC activation parameters as above. The resistance of hydrogels to bacterial biofilms was evaluated by disk method. Three kinds of bacteria (100 μL 120 CFU mL^-1^) were first inoculated on the surface of LB AGAR without any resistance and incubated in a 37°C constant temperature incubator for 12 hours. The formation of bacterial membrane was observed. At this time, the sterilized hydrogel was added and the bacterial membrane was incubated in a 37 °C constant temperature incubator for 12 hours. The area of inhibition zone was measured by ImageJ software.

**1.20 Western blot**

Western blot is used to quantitatively analyze and evaluate protein expression in the wound area. The wound tissue obtained was fully ground and broken by the grinder, and then lysate was added to the ice to extract protein. The 10% SDS-polyacrylamide gel electrophoresis gel then separates the same amount of protein from the tissue lysate and transfers it to the PVDF membrane. Primary antibodies against growth factor proteins, including VEGF and CD31, were added, followed by goat anti-rabbit IgG conjugated with horseradish peroxidase (HRP) (source: goat, species specific: rabbit, dilution 1:2000). Beta-actin (source: rabbit, species-specific: mouse, dilution 1:2000) was used as an internal control. Visualize protein analysis using quantitative software (Azure Biosystems C300, Azure C300, USA).

**1.21 Tissue staining**

The regenerated tissue of the infected wound on the 16th day was fixed with 4% paraformaldehyde. After paraffin embedding, the sample was crosscut into 5 μm thick slices and placed on the slide for staining. Histological analysis was then performed by heme-eosin (H&E) and Masson staining according to the manufacturer's instructions. At the same time, VEGF (GEM0022-48T) and CD31 (GB120005-100) immunofluorescence staining were performed using the standard protocol, and the levels of growth factor VEGF and CD31 in the regenerated tissues were detected by immunofluorescence staining of the tissue sections. In addition, western blot was used to detect the expression of VEGF and CD31-related proteins in the regenerated tissues of infected wounds in different groups. Finally, H&E staining was performed on the heart, liver, spleen, lungs, and kidneys to assess the in vivo toxicity of the hydrogel. All sections were photographed under a microscope.

**1.22 Immunofluorescence staining**

RAW264.7 cells were cultured in DMEM medium supplemented with 10% FBS according to standard procedures. Cells were seeded at 5×10⁴ cells/well into 24-well plates pre-incubated with sterile cell coverslips and cultured overnight. Each group was stimulated with LPS for 24 hours; the control group received only complete medium. Aspirate the medium and gently wash the cells twice with PBS. Add 4% polyformaldehyde (PFA) and fix at room temperature for 15 minutes. Discard the PFA and wash three times with PBS for 5 minutes each. Add 0.1% Triton X-100 and permeabilize at room temperature for 10 minutes to enhance antibody penetration. Wash three times with PBS. Add 10% normal goat serum and block at room temperature for 1 hour to reduce non-specific binding. Dilute primary antibodies (CD86 and CD206) according to manufacturer's instructions (1:200 dilution) and incubate overnight at 4°C in blocking solution. Discard primary antibody and wash three times with PBS for 5 minutes each. Add the corresponding fluorescently labeled secondary antibody (Alexa Fluor 488-conjugated anti-mouse IgG), incubate protected from light at room temperature for 1 hour. Optionally add DAPI for nuclear staining, incubate protected from light for 5 minutes. Mount slides with an anti-fluorescence quenching mounting medium. Observe and photograph under a fluorescence microscope or confocal microscope to document the localization and expression of CD86 (M1 marker) and CD206 (M2 marker).

**1.23 Transcriptome Analysis**

On day 16 post-injury, wound tissues were collected from infected diabetic mice and gently rinsed with sterile normal saline to remove blood and debris. Total RNA was extracted using TRIzol reagent (Invitrogen, USA) according to the manufacturer’s instructions. The quality of the extracted RNA was evaluated using an Agilent 5300 Bioanalyzer (Agilent Technologies, USA), and RNA concentration was measured using an ND-2000 spectrophotometer (NanoDrop Technologies, USA). All subsequent steps, including RNA purification, reverse transcription, library construction, and high-throughput sequencing, were performed by Shanghai Majorbio Bio-Pharm Technology Co., Ltd. (Shanghai, China) using the Illumina sequencing platform (Illumina, San Diego, CA, USA), following the standard protocols provided by the manufacturer. Differential gene expression analysis was conducted by quantifying transcript abundance using the Transcripts Per Million (TPM) method. Differentially expressed genes (DEGs) between sample groups were identified based on established statistical criteria. Functional annotation and enrichment analysis of the DEGs were performed using the Gene Ontology (GO) and Kyoto Encyclopedia of Genes and Genomes (KEGG) databases. Specifically, GO enrichment analysis was carried out using Goatools, while KEGG pathway enrichment was performed using KOBAS. Enriched GO terms and KEGG pathways were considered statistically significant when the Bonferroni-corrected P-value was ≤ 0.05, as compared to the background of the entire transcriptomic dataset.

**Figures S1–S21**

**
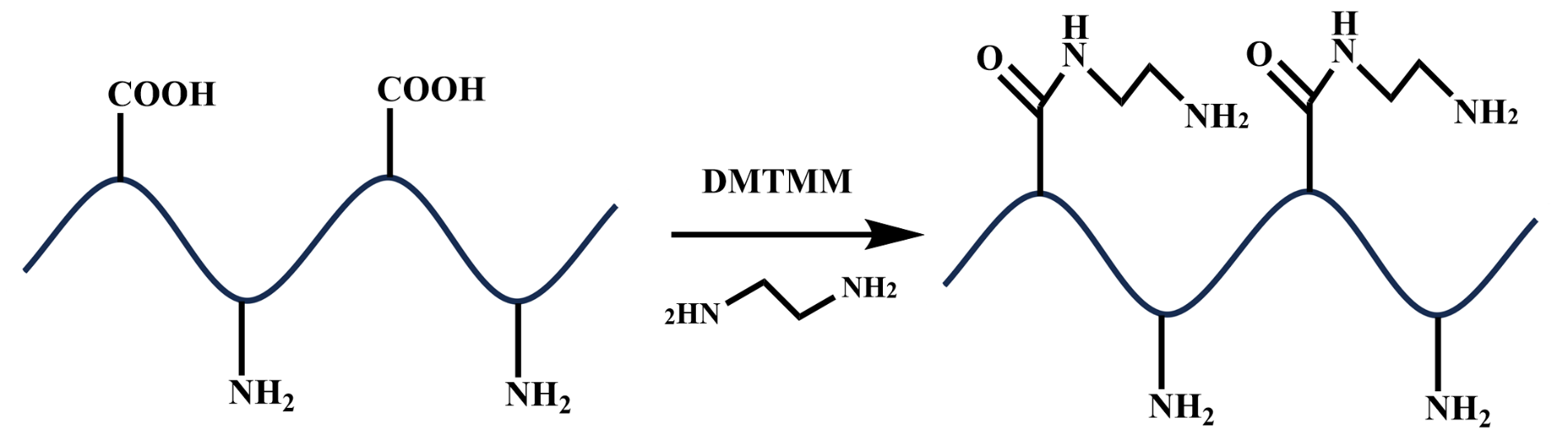
**

**Figure S1.** Chemical structural formula for the preparation process of amino gelatin.

**
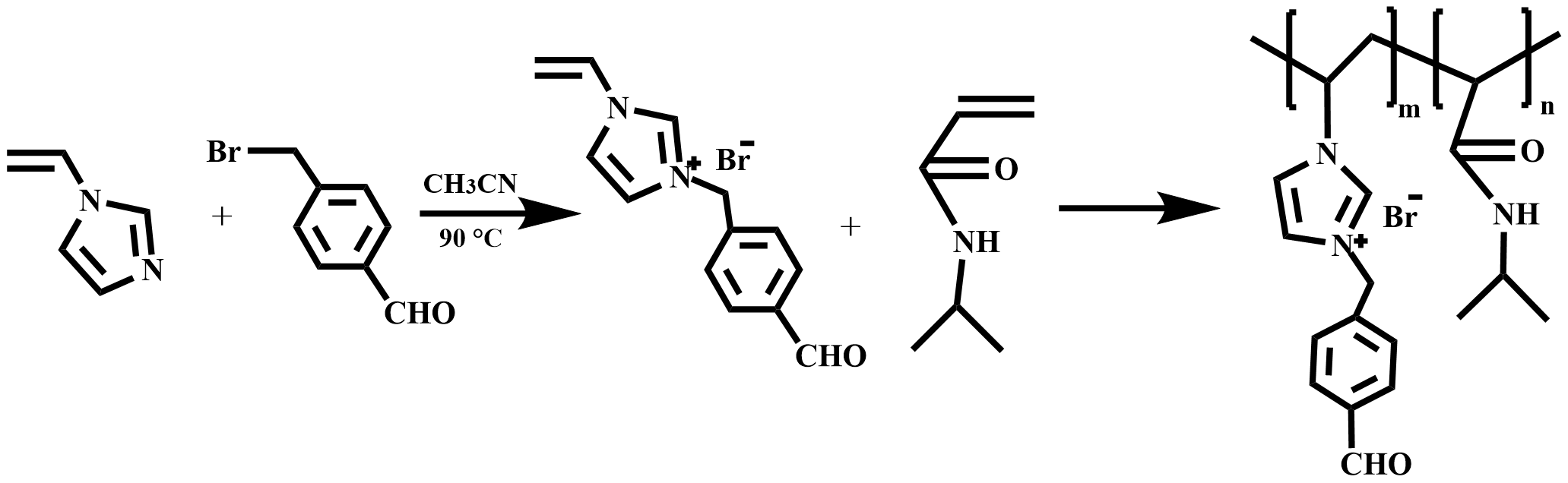
**

**Figure S2.** Crosslinking polymerization of poly ionic liquids with the temperature sensitizer NIPAM.





**Figure S3.** Fourier infrared spectra of gelatin and amino gelatin.


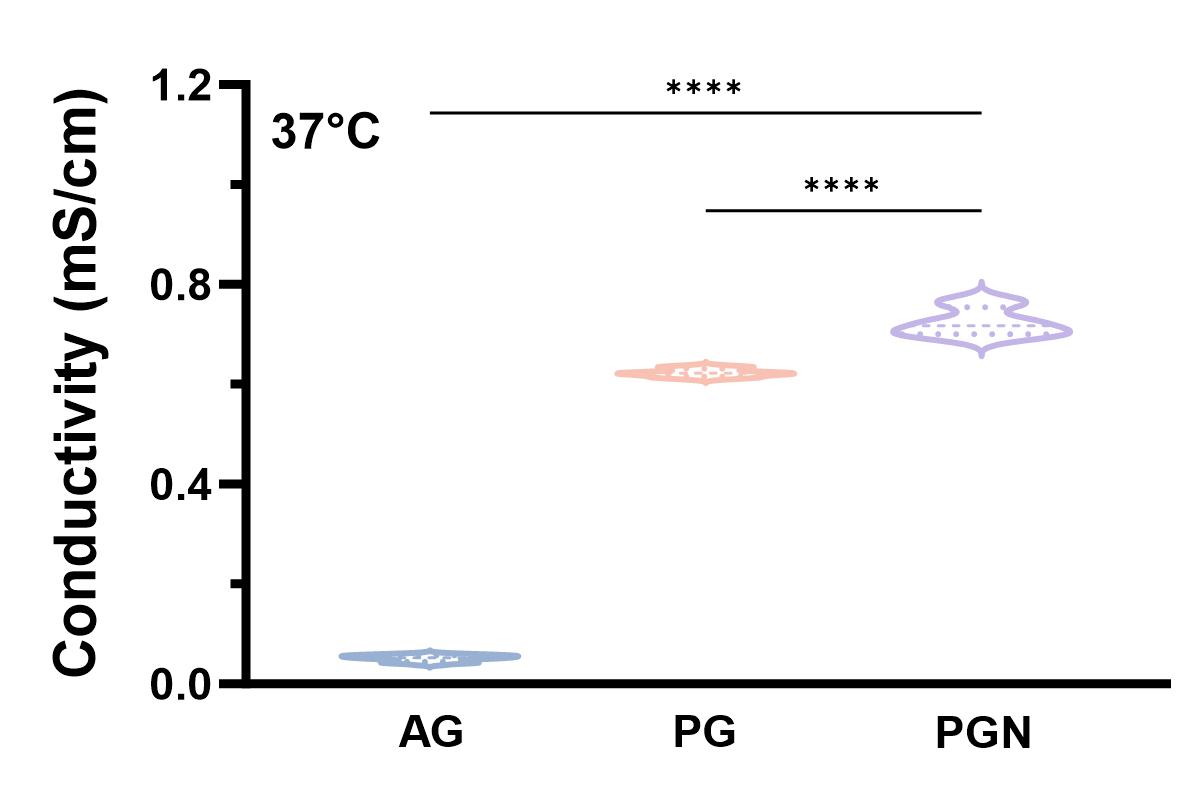


**Figure S4.** Conductivity statistics at 37 °C.


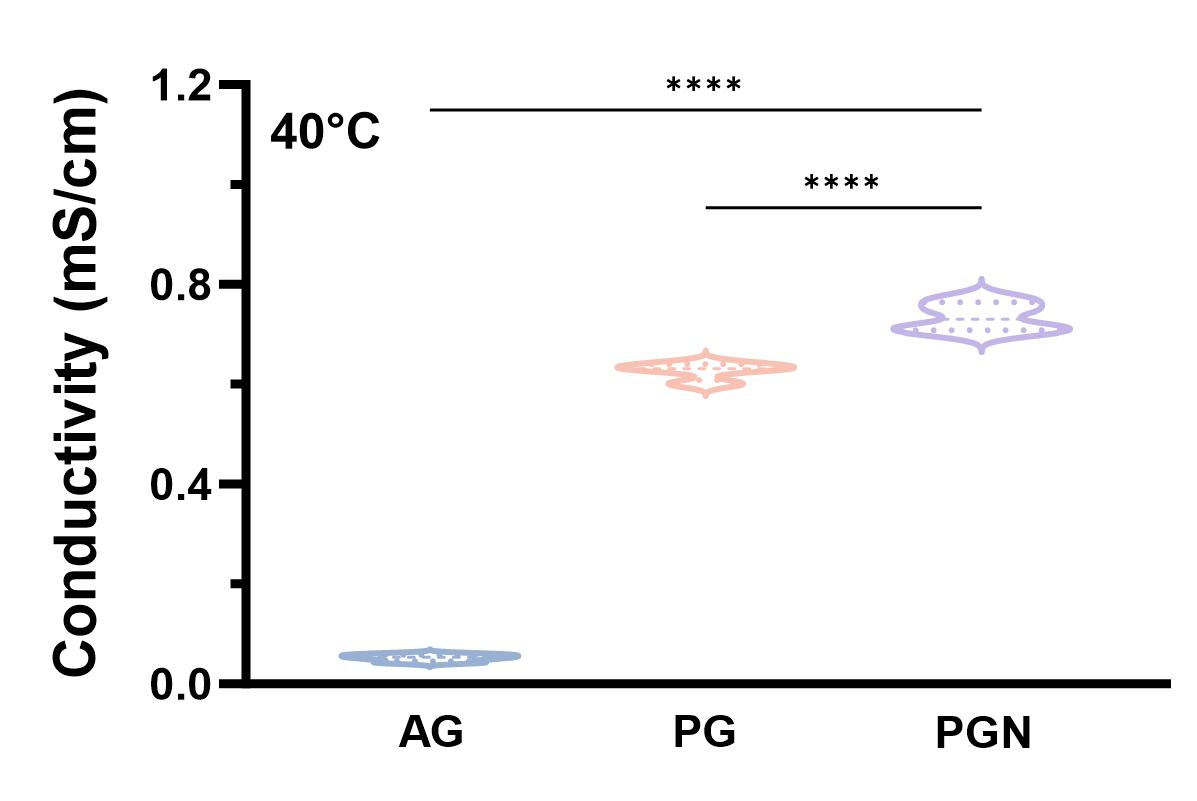


**Figure S5.** Conductivity statistics at 40 °C.


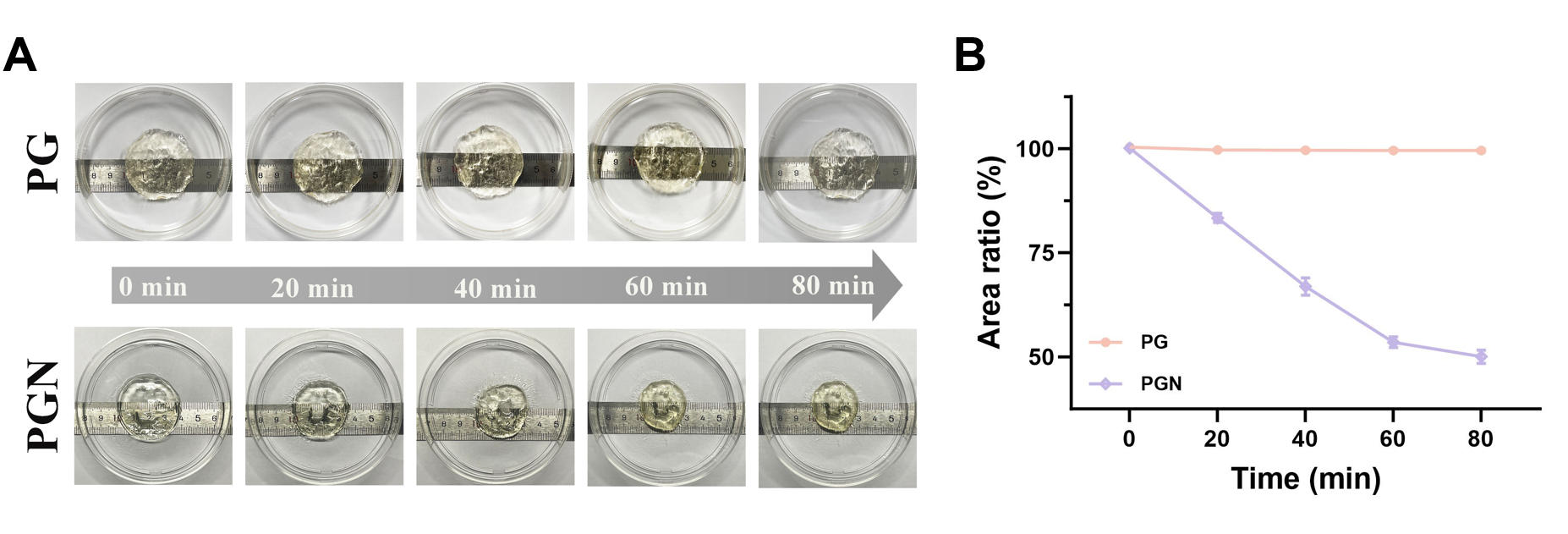


**Figure S6.** The area changes of PG and PGN hydrogels at 37 °C within 80 minutes. n = 3 independent samples, data represent mean ± SD; *p < 0.05, **p < 0.01, ****p < 0.0001.


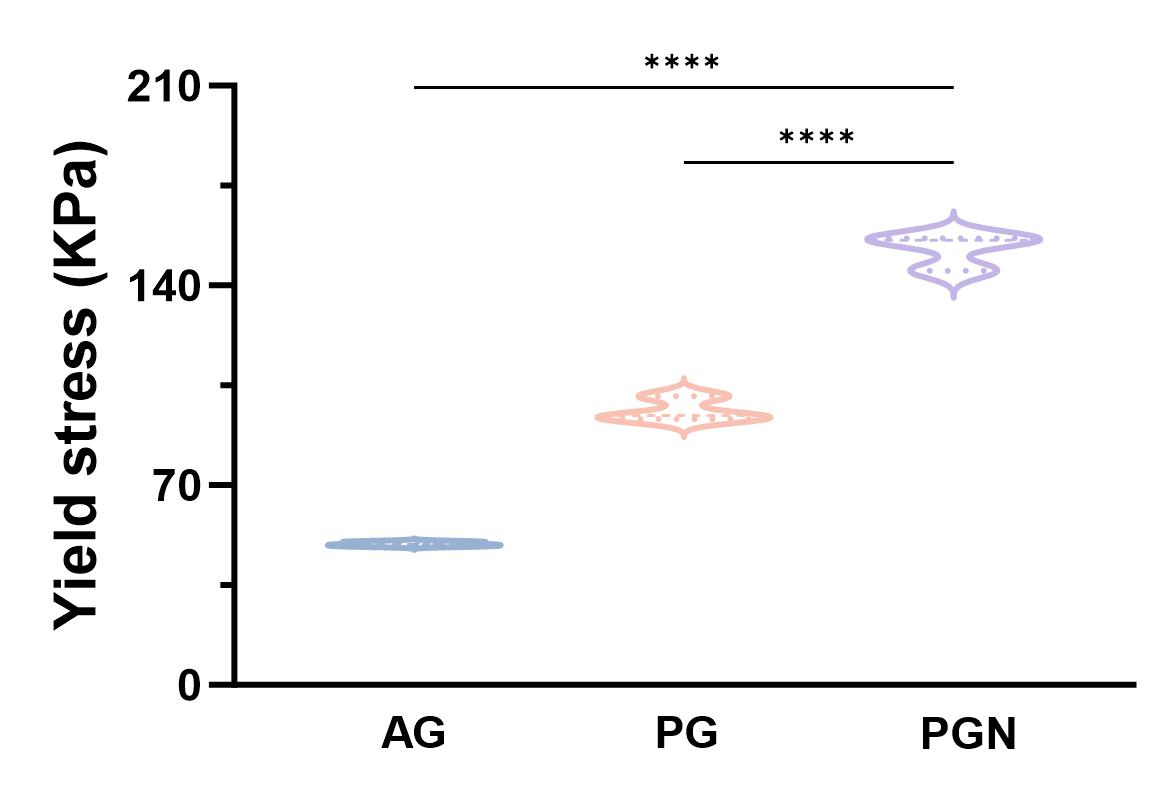
**Figure S7.** Yield stress at break of PGN, PG and AG hydrogels. n = 3 independent samples, data represent mean ± SD; *p < 0.05, **p < 0.01, ****p < 0.0001.


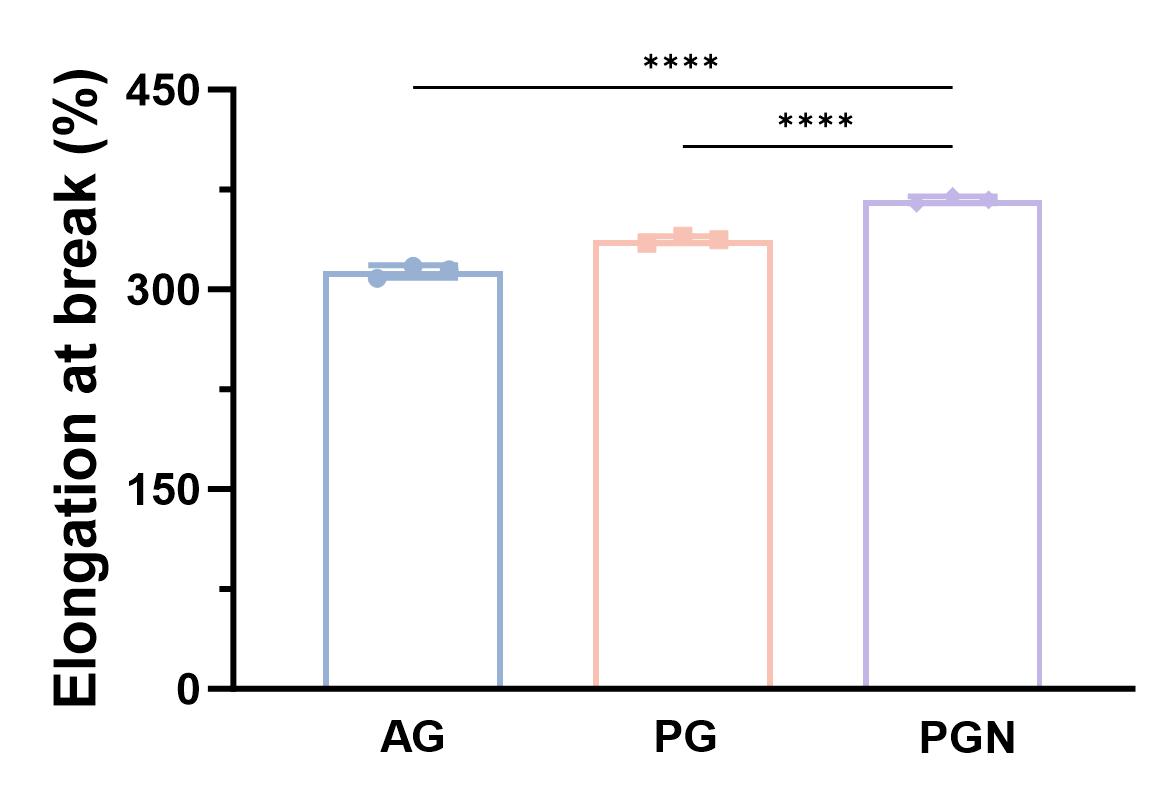
**Figure S8.** Elongation at break of PGN, PG and AG hydrogels. n = 3 independent samples, data represent mean ± SD; *p < 0.05, **p < 0.01, ****p < 0.0001.


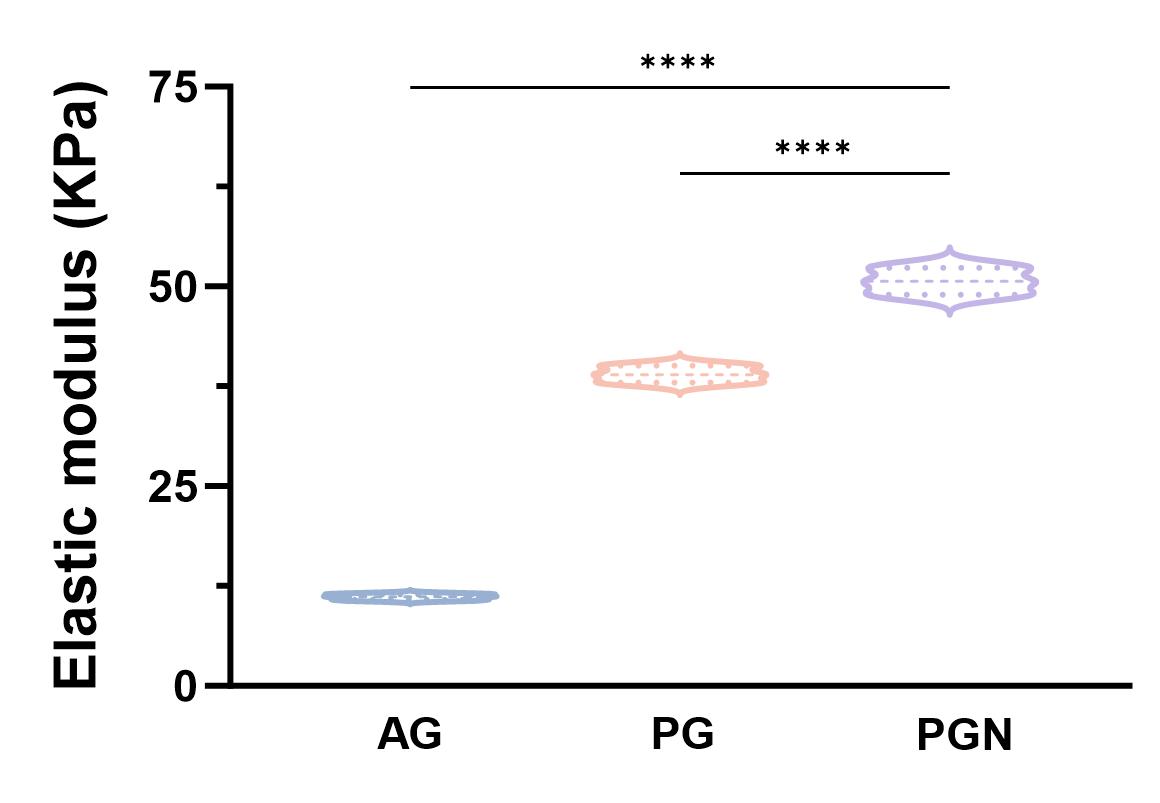


**Figure S9.** Elastic modulus of PGN, PG and AG hydrogels. n = 3 independent samples, data represent mean ± SD; *p < 0.05, **p < 0.01, ****p < 0.0001.


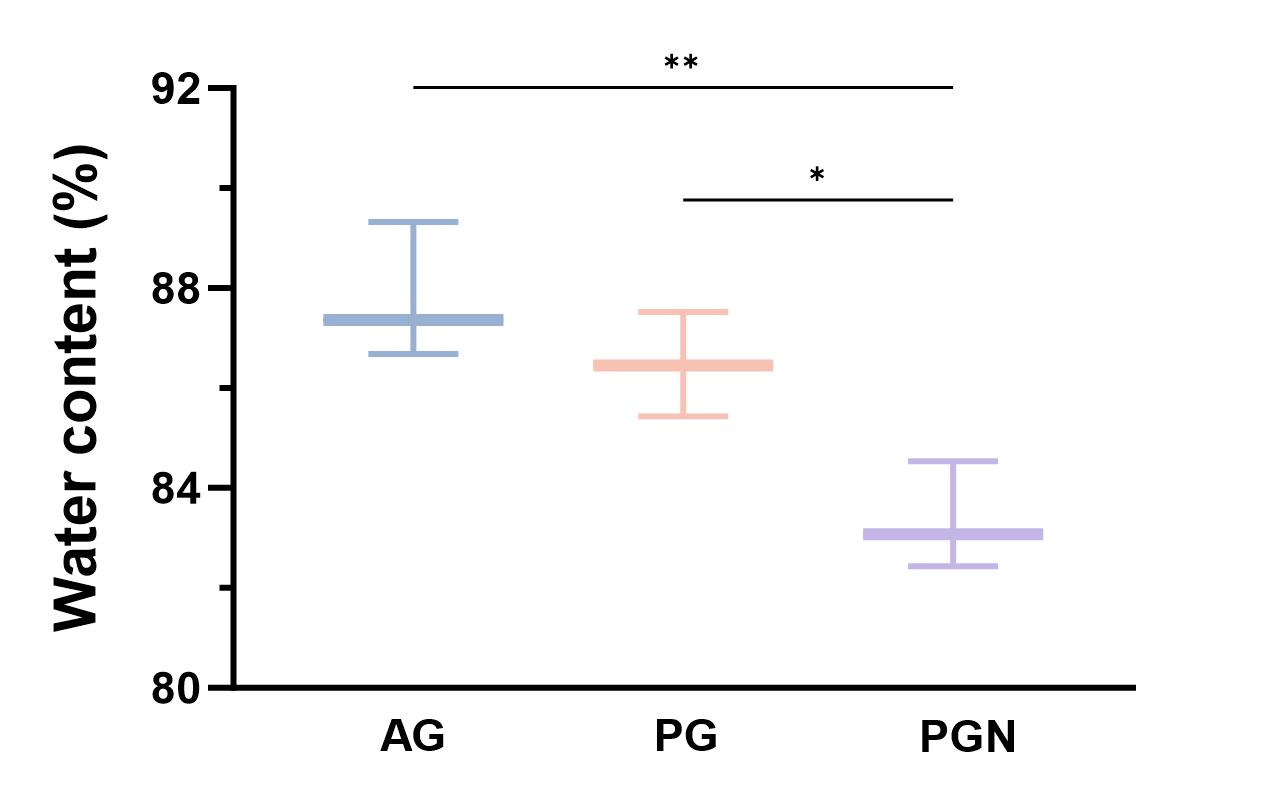
**Figure S10.** Water content of hydrogels. n = 3 independent samples, data represent mean ± SD; *p < 0.05, **p < 0.01, ****p < 0.0001.


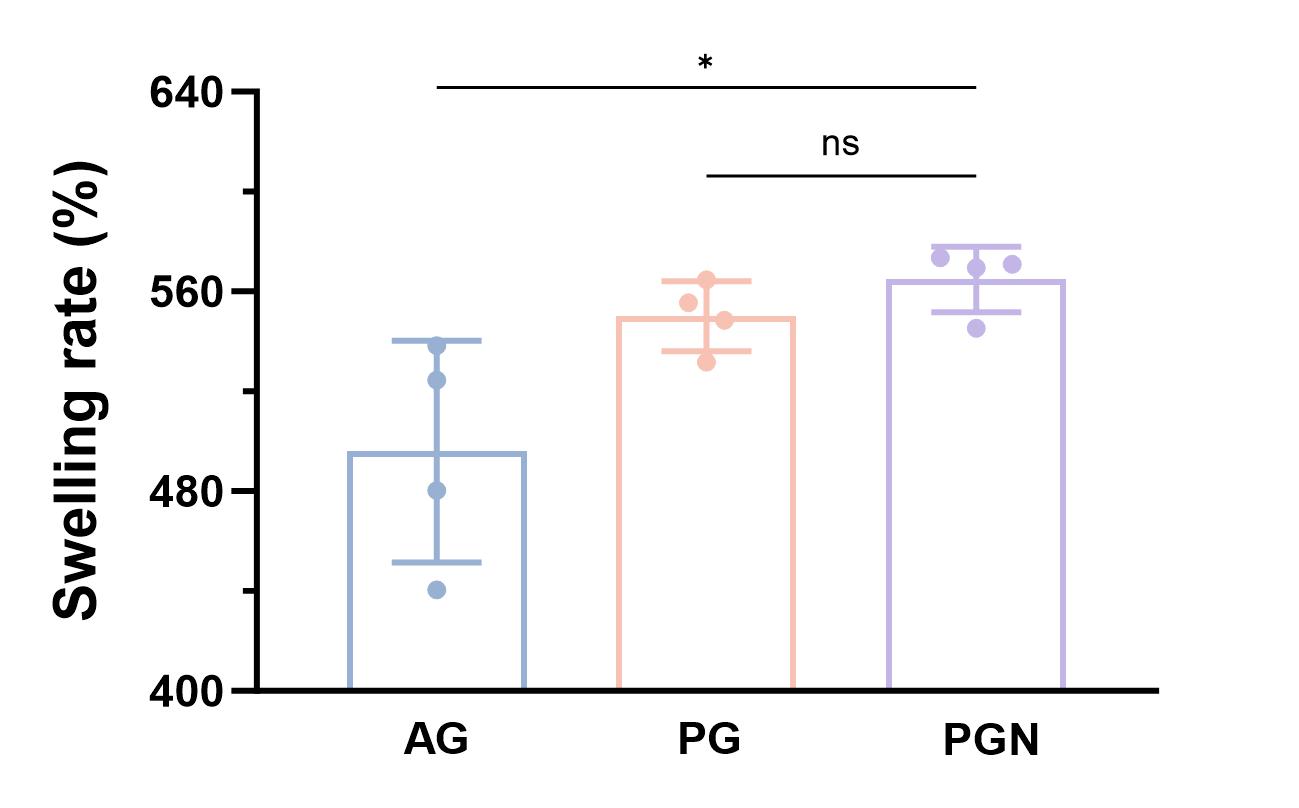


**Figure S11.** Swelling rate of hydrogels. n = 3 independent samples, data represent mean ± SD; *p < 0.05, **p < 0.01, ****p < 0.0001.


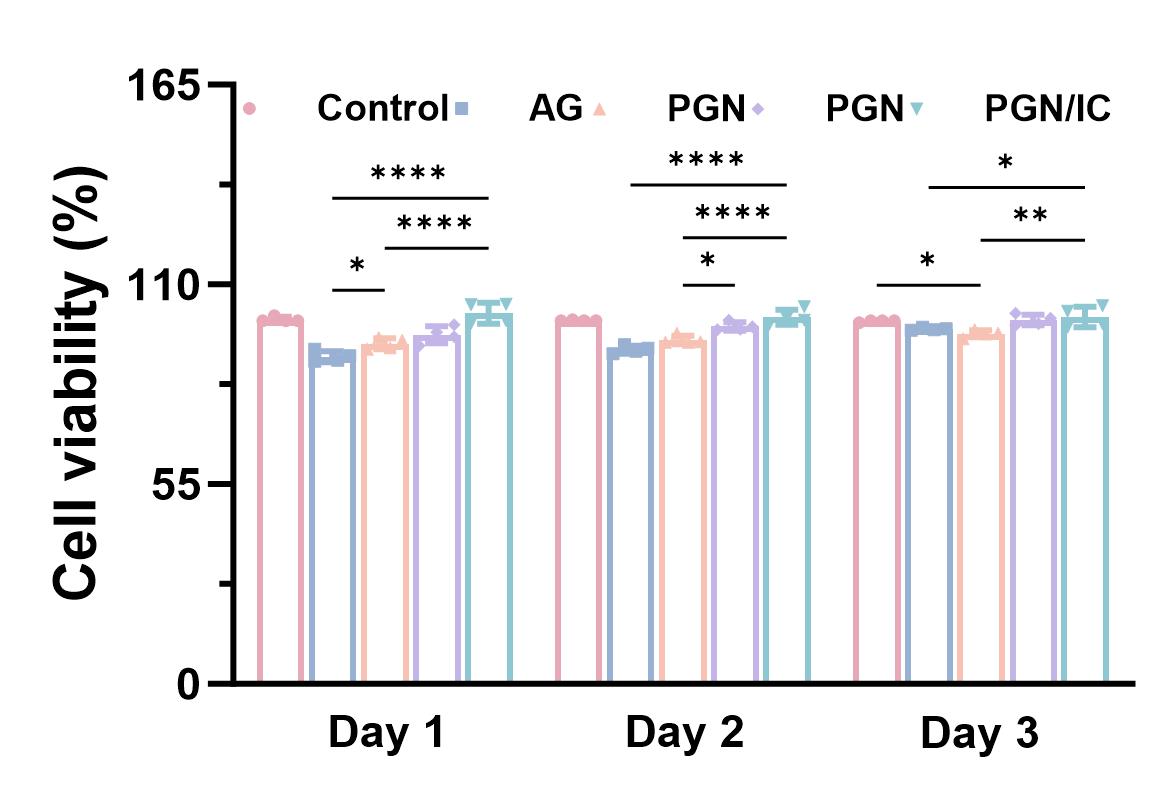


**Figure S12.** CCK-8 determination of L929 cells treated with different hydrogels for 1-3 days. n = 3 independent samples, data represent mean ± SD; *p < 0.05, **p < 0.01, ****p < 0.0001.


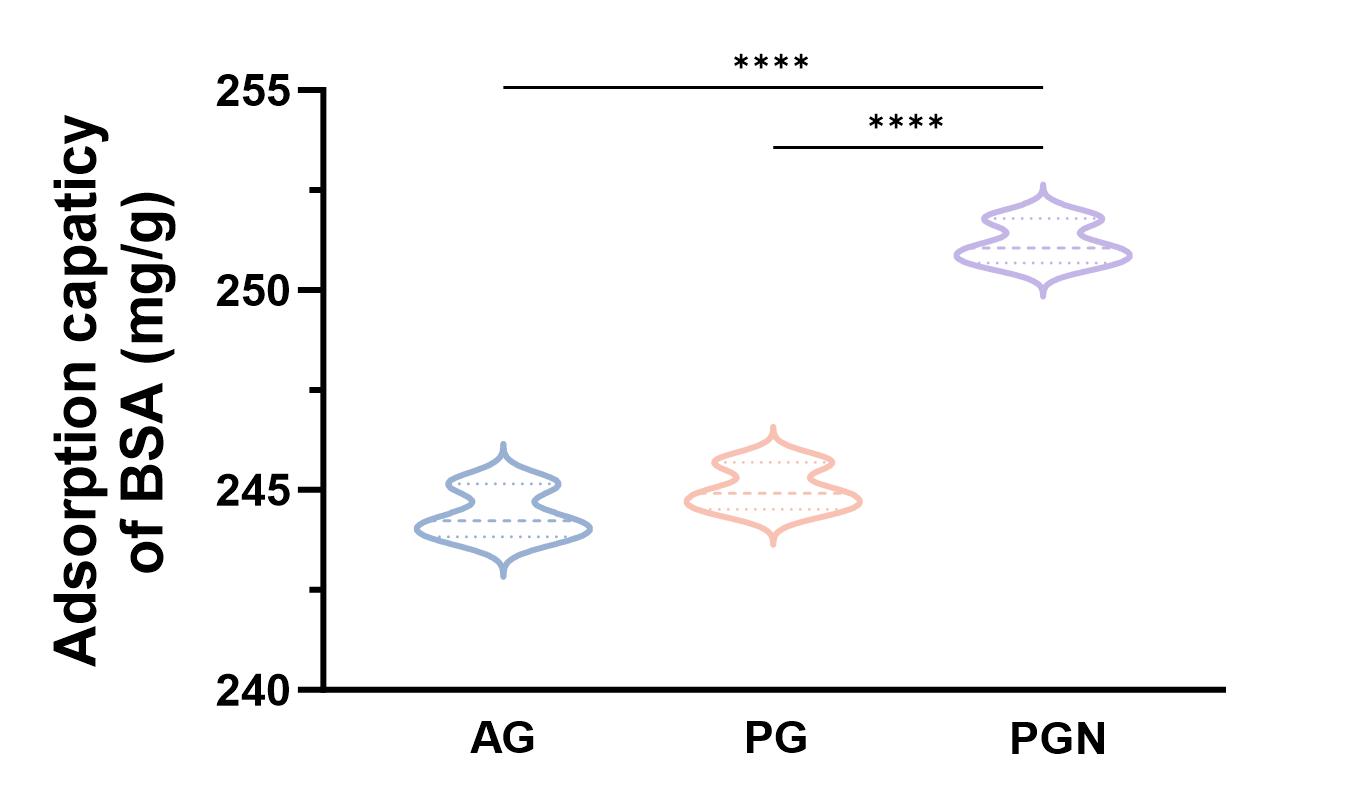


**Figure S13.** Adsorption properties of a series of hydrogels for BSA . n = 3 independent samples, data represent mean ± SD; *p < 0.05, **p < 0.01, ****p < 0.0001.


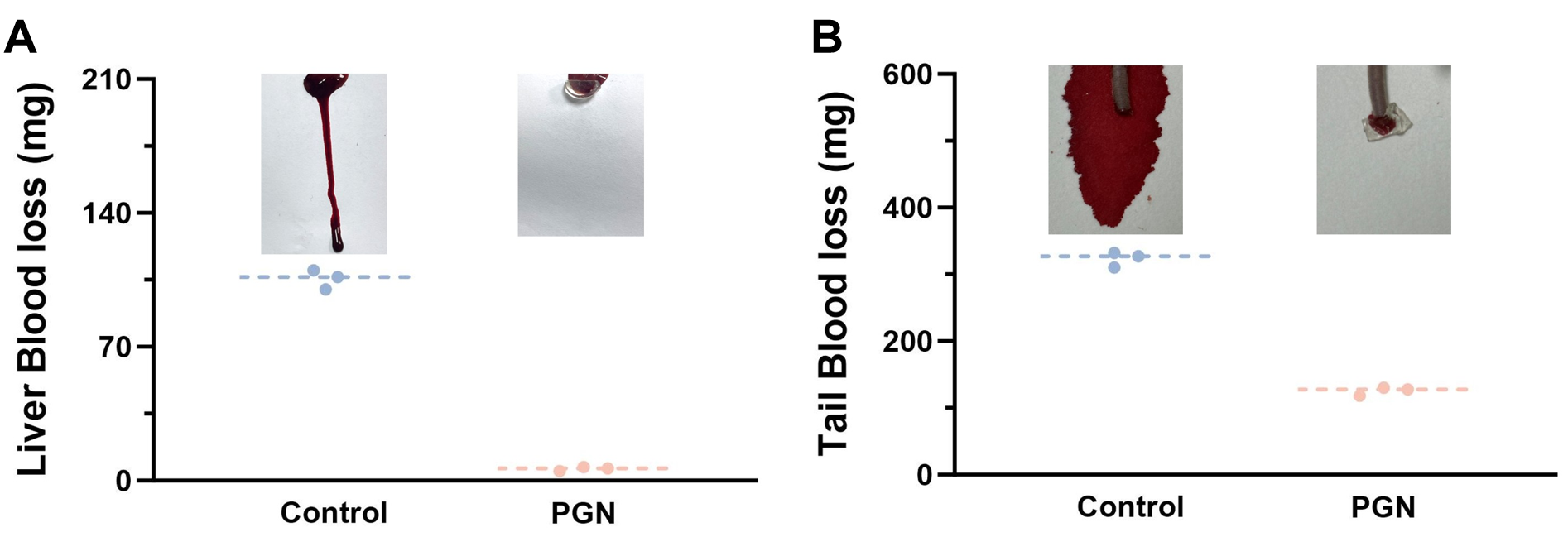


**Figure S14.** Hemostatic properties of hydrogels at tail and liver sites. n = 3 independent samples, data represent mean ± SD; *p < 0.05, **p < 0.01, ****p < 0.0001.


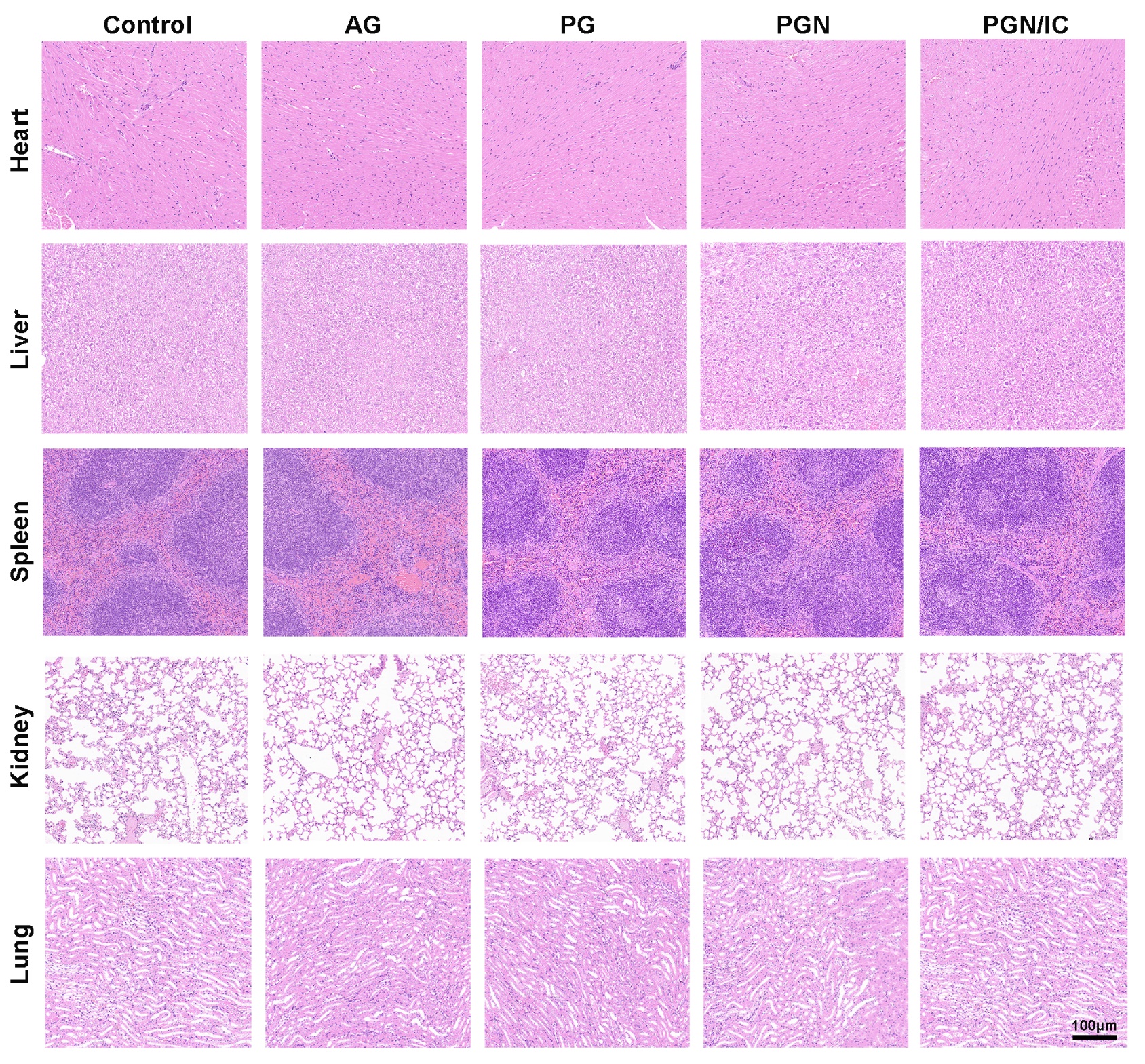


**Figure S15.** H&E staining images of heart, liver, spleen, lungs and kidneys of db/db mice treated with different materials. Scale bar :100 μm.


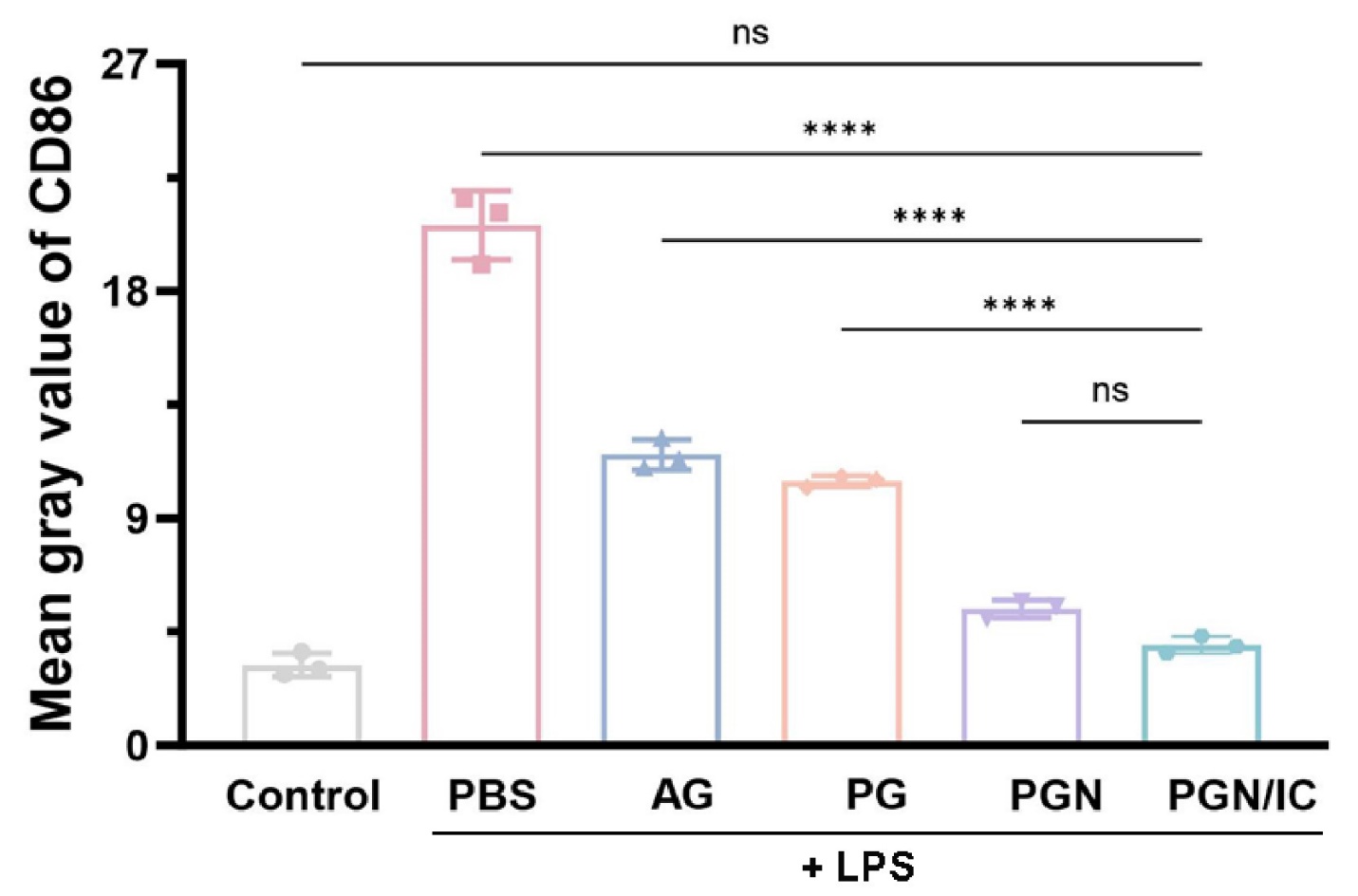
**Figure S16.** Fold change in CD86 fluorescence intensity; n = 3 independent samples, data represent mean ± SD; *p < 0.05, **p < 0.01, ****p < 0.0001.


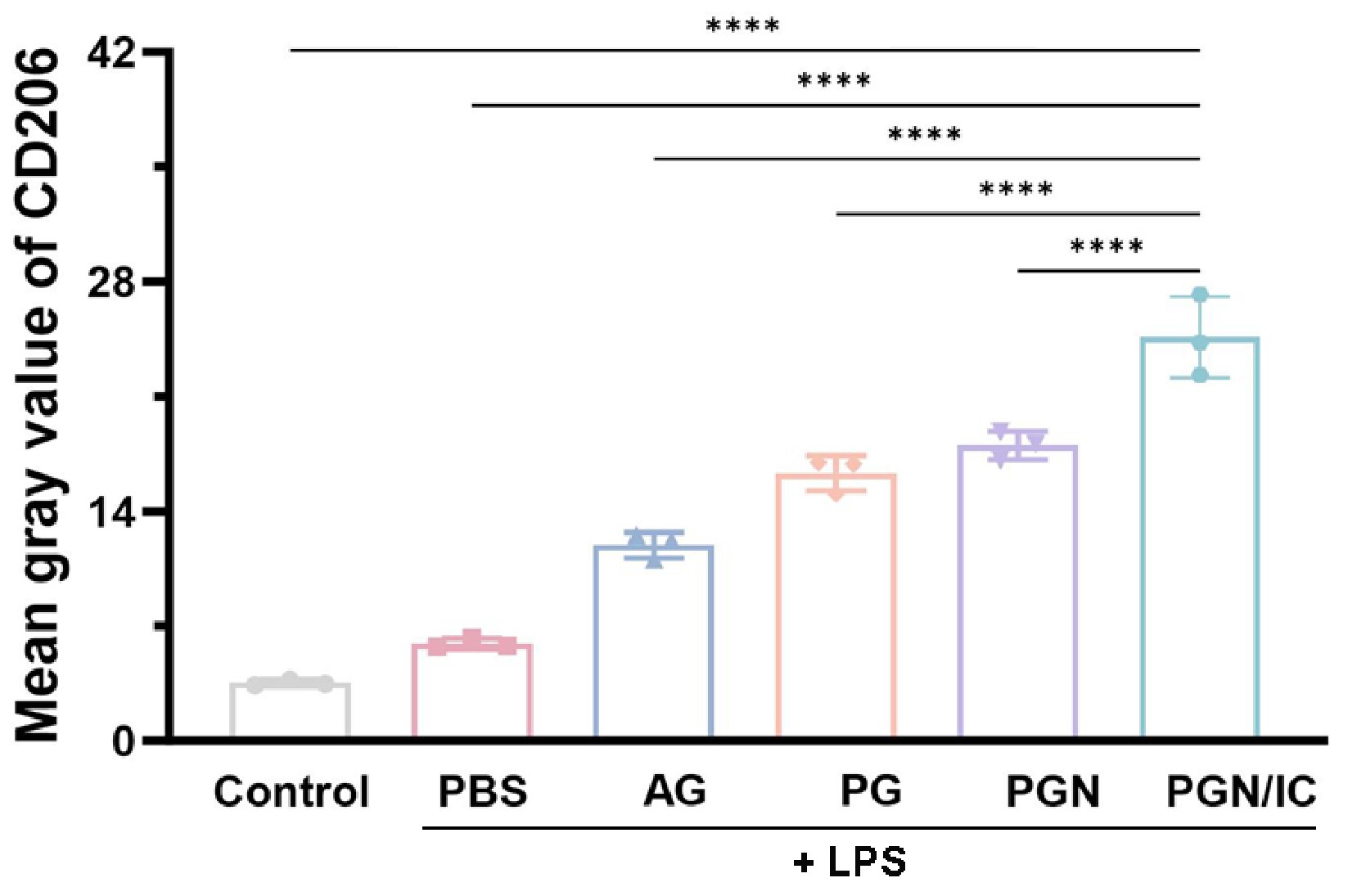
**Figure S17.** Fold change in CD206 fluorescence intensity; n = 3 independent samples, data represent mean ± SD; *p < 0.05, **p < 0.01, ****p < 0.0001.





**Figure S18.** Relationship between receiver and transmitter in wireless power supply.





**Figure S19.** Wireless transmission distance.


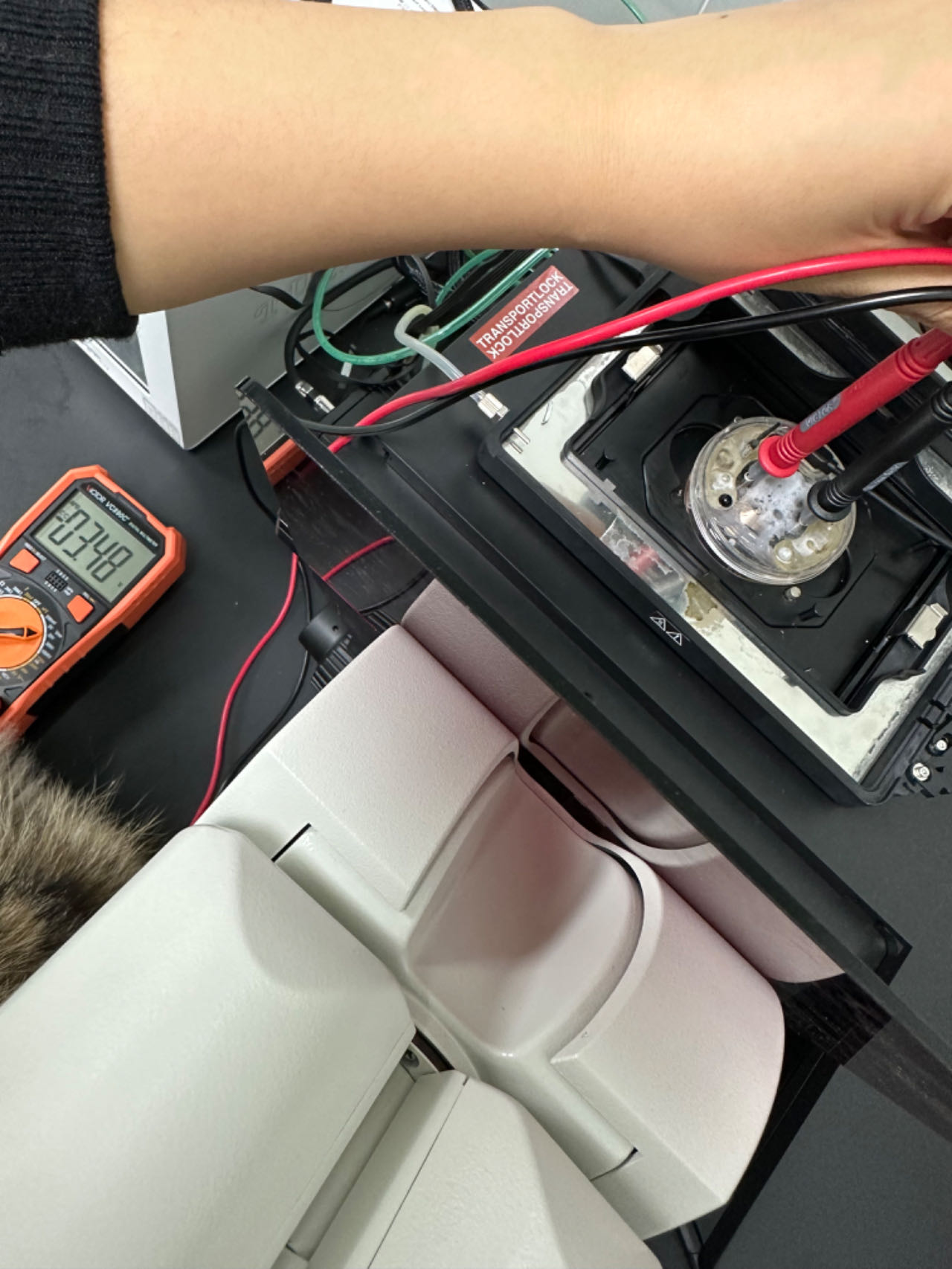


**Figure S20.** Measure the electric field in the medium.


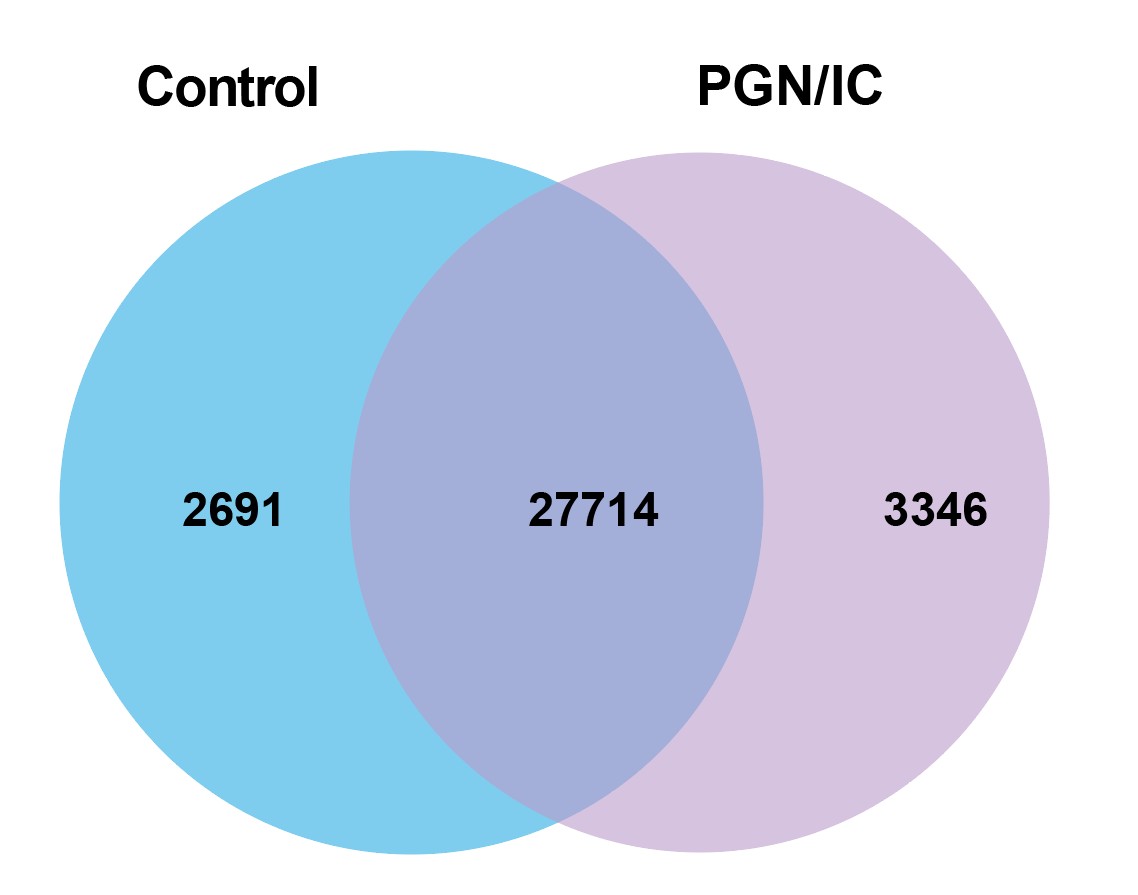


**Figure S21.** Venn diagram of the differentially expressed genes between Control and PGN/IC.

[1] Y. G. Zong, P. Liu, R. Zha, B. Zong, Y. Wang, H. Fang, W.-L. Wong,C. Li, Sensors and Actuators B: Chemical **2023**, 394, 23.

[1] Y. G. Zong, B. Zong, R. Zha, Y. Zhang, X. Li, Y. Wang, H. Fang, W. L. Wong,C. Li, Adv. Healthc. Mater. **2023**, 12, 2301245.
